# Porous Ti-6Al-4V Architectures in Load-Bearing Orthopedic Reconstruction: A Critical Narrative Review of the Translational Gap Across Microstructure, Fatigue, Surface Function, and Clinical Failure Modes

**DOI:** 10.3390/ma19183860

**Published:** 2026-09-10

**Authors:** Gündüz Ercan Kutluay, Fatih Erdoğan, Yaşar Mahsut Dinçel

**Affiliations:** 1 Department of Orthopedics and Traumatology, ÇOSB Kapaklı State Hospital, 59500 Tekirdağ, Türkiye; 2 Department of Orthopedics and Traumatology, Faculty of Medicine, Tekirdağ Namık Kemal University, 59030 Tekirdağ, Türkiye; op.dr.fatiherdogan@gmail.com (F.E.); ymd61@gmail.com (Y.M.D.)

**Keywords:** porous titanium, Ti-6Al-4V, additive manufacturing, β-titanium, fatigue, osseointegration, stress shielding, load-bearing orthopedic implant

## Abstract

An aging population and a rising revision burden are increasing demand for bone-compatible load-bearing implants. Because the elastic modulus of conventional Ti-6Al-4V (~110 GPa) exceeds that of cortical bone (7–30 GPa), stress shielding can drive bone resorption and aseptic loosening. Two solution lines have emerged: low-modulus β-type alloys and porous architectures. Focusing on additive manufacturing (AM), this critical narrative review synthesizes the evidence along the axis of clinical failure modes. Materials science has lowered β-Ti’s modulus to ~40 GPa in bulk, yet clinical implants rely predominantly on porous conventional Ti-6Al-4V: the low effective modulus (single-digit GPa) comes from architecture, not alloying. These architectures range from porous fixation surfaces on solid acetabular shells to predominantly porous constructs—revision knee cones and sleeves, acetabular augments, and spinal interbody cages. In the acetabular cohorts, where clinical evidence is concentrated, short- to mid-term survivorship is favorable though heterogeneous; revisions were driven mainly by infection and instability, with aseptic loosening low. Two tools are proposed: a synthesis matrix setting laboratory claims alongside clinical evidence for each design parameter, and a 16-item minimum reporting checklist. Applied to the 12 primary series reviewed, the checklist found manufacturing and architecture-verification parameters largely unreported even where clinical outcomes are well documented.

## 1. Introduction

Metallic biomaterials are the foundation of modern orthopedic surgery, valued for their high specific strength, corrosion and fatigue resistance, and superior biocompatibility [1]. Among stainless steels, cobalt-based alloys, and titanium alloys, titanium is increasingly the material of choice for joint prostheses, spinal fusion devices, and trauma implants, thanks to its low density, excellent biocompatibility, and well-characterized clinical track record. These material groups also differ in how fixation is achieved. Stainless steel and the majority of cobalt–chromium components are used with cement fixation, in which polymethylmethacrylate provides immediate mechanical interlock between implant and bone. Titanium alloys, by contrast, are used predominantly in cementless designs that rely on direct bone apposition and ingrowth for durable fixation. Throughout this review, osseointegration denotes the direct structural and functional connection between ordered, living bone and the surface of a load-bearing implant, without an intervening fibrous layer. This distinction frames what follows: porous architecture is a strategy for biological rather than cemented fixation, and its rationale, its advantages, and its failure modes are accordingly those of cementless, ingrowth-dependent implants. As the population ages and primary arthroplasty volumes grow, the revision burden is climbing rapidly: revision total hip arthroplasties (rTHAs), for example, are projected to rise by 43–70% by 2030 (as reported by Berlinberg et al. [2]). The scale is striking—in the United States alone, roughly 790,000 total knee and 544,000 total hip arthroplasties are performed each year, and about one-third of adults over 40 carry at least one metal implant (as compiled by McCloskey et al. [3])—sharpening the need for implants that are not only durable but also mechanically and biologically matched to the surrounding bone.

A major biomechanical limitation of titanium alloys is elastic modulus mismatch. Cortical bone has an elastic modulus of roughly 7–30 GPa and cancellous bone 0.3–4 GPa, whereas conventional Ti-6Al-4V sits at 110–114 GPa, stainless steel at ~200 GPa, and Co-Cr alloys higher still [1]. Because of this mismatch, the implant carries a disproportionate share of the load, reducing the physiological load transfer and mechanical stimulus to the surrounding bone, which may, over time, undergo resorption, loosening, and secondary fractures—a process known as “stress shielding”. The clinical consequences of this process extend beyond aseptic loosening: periprosthetic bone resorption has been identified as an independent risk factor for postoperative periprosthetic femoral fracture [4], and the associated loss of proximal bone stock complicates any later reconstruction, making revision surgery technically more demanding [5]. The clinical evidence for these consequences—and its limits—is examined in Section 7.1. Low-Young’s-modulus β-type titanium alloys were developed specifically to counter this bone atrophy while retaining adequate static and dynamic strength [6]. A further concern is the long-term release of potentially toxic aluminum and vanadium from Ti-6Al-4V [1]. This concern has not, however, translated into a demonstrated clinical liability for load-bearing implants: the extra-low-interstitial grade (Ti-6Al-4V ELI, Grade 23) is used in most such devices, and decades of arthroplasty follow-up have not established Al/V release as a clinically significant failure driver at the systemic level. Ion release, therefore, remains a materials-level motivation for alternative chemistries rather than a settled clinical problem—a tension this review returns to when weighing the two solution lines.

This qualification applies to the bulk of the implant and to systemic ion release; it does not extend to modular junctions. Where a stem must articulate with a femoral head, the head-neck taper forms a crevice subject to micromotion under cyclic load, and mechanically assisted crevice corrosion at that junction—commonly termed trunnionosis—is a recognized clinical entity that can release metal debris and ions locally, provoke adverse local tissue reactions, and lead to revision [7]. Its severity is governed by junction design and assembly rather than by bulk alloy chemistry: taper geometry and engagement, neck and stem flexural rigidity, head size, the material couple across the junction, and the assembly conditions [7,8]. Two implications follow for this review. First, the porous architectures examined here are applied to fixation surfaces, not to modular or articulating interfaces, which remain solid by design—one of the reasons clinically translated devices combine a solid structural substrate with a porous interface. Second, and less comfortably, any strategy that lowers the structural stiffness of a stem, whether by alloy chemistry or by porous architecture, increases its elastic deformation under load and could, in principle, increase micromotion at a modular junction; this interaction has not been characterized for fully porous stems and is one of the open questions carried into the fretting and corrosion-fatigue discussion of Section 5.4.

Two main solution lines have matured in the literature. The first is the design of low-modulus β-type titanium alloys using biocompatible β-stabilizing elements (Nb, Ta, Zr, Mo, and Sn) to lower the material’s intrinsic elastic modulus. Which of these alloys have actually reached commercial implants—and why most have not—is taken up at the end of Section 3.2. The second is to reduce the structure’s effective modulus through porous architectures—independent of alloy composition—while providing a surface favorable for bone ingrowth. Both approaches have advanced considerably over the past two decades.

The central observation of this review, however, is the pronounced gap between these two solution lines and clinical practice. In the laboratory, materials science has lowered the elastic modulus of β-type alloys to only ~40 GPa in bulk form and to single-digit GPa values in porous form. Yet most load-bearing implants that have reached the market and entered widespread use are based on the porous architecture of conventional Ti-6Al-4V rather than on these low-modulus β-Ti alloys. The porous regions of clinical acetabular cups, for instance, reach a low effective modulus (~0.5–3 GPa) through structure rather than through alloying—this being the modulus of the porous architecture itself, not the overall stiffness of the whole component. This review, therefore, asks why the gap between the low-modulus alloys developed and extensively characterized in the laboratory and the solutions actually used in the clinic exists and what this implies for porous titanium implant design.

To answer these questions, this review makes three main contributions that set it apart from the existing literature. First, it organizes the clinical evidence by clinical failure mode rather than by implant type, tying each material or design decision directly to the failure mode it is meant to address. Second, for the principal design parameters, it places the laboratory claim and the clinical evidence side by side in a synthesis matrix. The purpose of this matrix is to separate two questions that are usually merged: what laboratory evidence claims a design parameter should achieve, and what clinical evidence actually demonstrates for it. Setting the two side by side for each parameter allows the strength of a design rationale to be judged independently of the strength of the clinical evidence supporting it, and makes the points of divergence explicit—those parameters for which a well-supported laboratory expectation has, as yet, no clinical correlate. Third, it proposes an original minimum reporting/standardization checklist to address the reporting inconsistencies that pervade porous titanium implant studies. Recent reviews of porous titanium in orthopedics (e.g., [3,9]) offer valuable syntheses across design, biofunctionalization, and patient-specific application but do not organize the clinical evidence by failure mode, place laboratory rationale and clinical outcome side by side for each design parameter or propose a failure-mode-linked reporting standard—the gap these three contributions are built to fill. The scope is limited to load-bearing orthopedic reconstruction, keeps an additive-manufacturing focus, and excludes dental applications and routine fracture fixation, since porous titanium is a defect/reconstruction biomaterial, whereas fracture fixation relies on solid plates, nails, and screws; post-traumatic or oncologic segmental defect reconstructions are included where a load-bearing porous titanium architecture is central to the implant. Across the applications examined, the maturity of clinical evidence varies widely: hip-acetabular reconstruction is the most mature area, backed by cohort-level survivorship data, and forms the main axis of the review’s clinical evidence, whereas spinal fusion, knee revision, and tumor/segmental reconstruction remain mostly at the level of case-series, biomechanical, or preclinical evidence—an asymmetry itself treated as a finding that supports the review’s laboratory–clinical gap thesis.

The remainder of the review is organized as follows: The methodology and literature search strategy are presented in Section 2; the rationale for porosity and titanium, together with low-modulus β-Ti alloys, is found in Section 3; advanced manufacturing methods are provided in Section 4; microstructure and fatigue behavior are offered in Section 5; surface engineering and osseointegration/antibacterial strategies are presented in Section 6; and clinical translation along the axis of failure modes is provided in Section 7. Section 8 covers challenges, future directions, and the proposed standardization checklist; Section 9 discusses the review’s limitations; and Section 10 concludes the synthesis.

## 2. Materials and Methods: Literature Search Strategy

This is a critical narrative review rather than a systematic review or meta-analysis, and the PRISMA protocol was, therefore, not applied; the terms “identified”, “screened”, and “included” are used only to describe the conceptual selection flow, not a formal PRISMA procedure, and the resulting evidence base is intended as an illustrative, thesis-driven synthesis rather than an exhaustive enumeration. The literature was searched in a targeted manner, primarily through PubMed/MEDLINE and Google Scholar—the latter used chiefly for backward and forward citation chasing rather than for reproducible enumeration—supplemented by publisher platforms (e.g., ScienceDirect and SpringerLink) and open-access journal databases. Searches combined key terms such as “porous titanium”, “Ti-6Al-4V”, “additive manufacturing”, “beta titanium/β-titanium”, “low modulus”, “stress shielding”, “osseointegration”, “acetabular reconstruction”, “fatigue”, and “periprosthetic joint infection”. The final search was run in June 2026. A representative PubMed string was (“porous titanium” OR “Ti-6Al-4V”) AND (“additive manufacturing” OR “3D printing”) AND (orthopedic OR acetabular OR arthroplasty OR spine OR scaffold).

The recent literature (predominantly 2018–2026) was prioritized, while foundational and landmark studies were also included. The inclusion criteria were material, manufacturing, surface, or clinical studies published in English or Turkish and relevant to load-bearing orthopedic reconstruction, with particular priority given to clinical cohort, case-series, and survivorship data on porous titanium implants. The exclusion criteria were dental applications (a non-articular, generally lower-load environment with different bone quality), routine fracture fixation (trauma), non-load-bearing applications, and pure materials-science studies without an orthopedic context, with the scope logic for fracture fixation and segmental defects, as set out in Section 1. Among clinical studies, those reporting cohort, survivorship, and patient-reported outcome (PROM) data on porous titanium load-bearing implants were prioritized, while those with only biomechanical or in vitro data were used contextually.

Citation integrity was maintained throughout: every cited source was examined, and any primary numerical data that could not be accessed directly and were instead taken from review sources are flagged explicitly in the text with the phrase “as compiled by …”.

**Figure 1:**
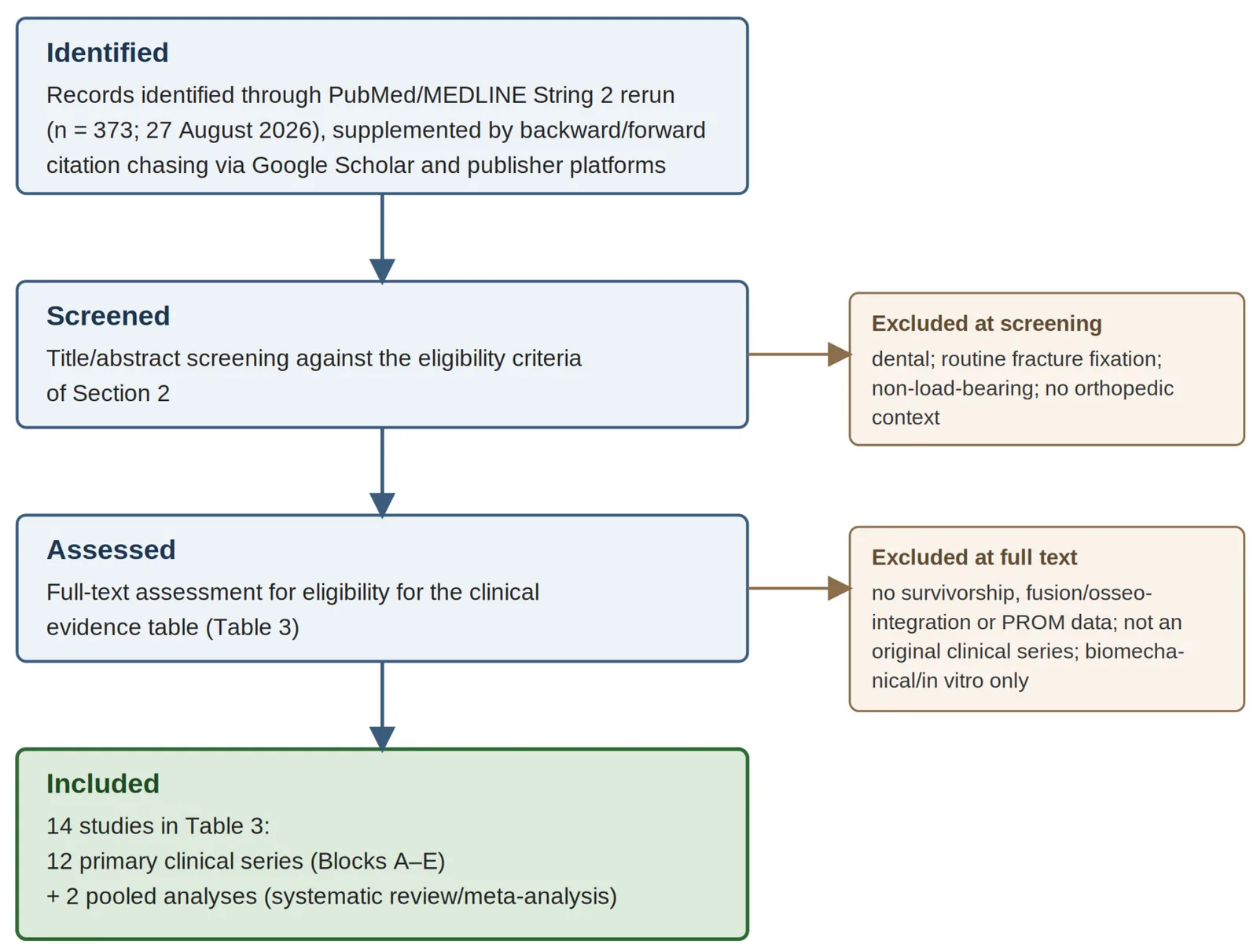
Study-selection flow for the clinical evidence base (Table 3). The identification count derives from the search-string rerun reported in Section 2.1. This is not a PRISMA flow diagram: consistent with the narrative design of the review, the figure records the provenance of the evidence actually used rather than stage-level record counts.

To keep the type and level of evidence transparent, the findings discussed here were weighed within an implicit evidence hierarchy based on source type. At the top are systematic reviews/meta-analyses and cohort-level clinical survivorship series, followed by case series, then controlled preclinical (in vivo animal) studies, and, finally, mechanistic inferences from in vitro, biomechanical, and finite-element analysis (FEA). Throughout, we aimed to indicate the evidence level on which each finding rests, keeping clinical survivorship data deliberately separate from mechanistic or preclinical inference. To qualify for the clinical evidence table (Table 3), a study had to be an original clinical series—or a meta-analysis pooling such series—in which a porous and/or additively manufactured titanium load-bearing implant was used in humans and that reported at least survivorship, fusion/osseointegration, or PROM data; purely technical or biomechanical reports were kept out of the table and used only contextually. Materials-science and manufacturing studies were chosen by prioritizing those with a direct load-bearing orthopedic connection (Ti-6Al-4V or biomedical β-Ti alloys; porous/lattice architecture; and the AM process–microstructure–fatigue relationship). Conceptually, the process ran in the following three stages: identifying the relevant literature through searching (identified); screening for those within scope at the title/abstract level (screened); and allocating suitable studies to either the clinical synthesis (Section 7) or the mechanistic/materials synthesis (Section 3, Section 4, Section 5 and Section 6) (included). Study identification and eligibility screening were carried out by two authors (G.E.K. and F.E.), with inclusion uncertainties resolved by discussion. Because this is not a systematic review, no formal record count was maintained during the original search, and the selection was purpose-driven, prioritizing the studies most informative for each failure mode rather than exhaustive capture—a deliberate limitation of the narrative format revisited in Section 9. To make the search auditable nonetheless, the core search strings are reported in full in Section 2.1, and the selection flow for the clinical evidence base (Table 3) is summarized—without claiming PRISMA compliance—in Figure 1.

Because the maturity of the clinical evidence varies markedly by application region (the most mature data being concentrated in hip-acetabular reconstruction), the clinical studies are structured along the axis of failure modes (Section 7) and in a separate evidence table (Table 3); this table is an illustrative, non-exhaustive synthesis of representative series rather than a systematic enumeration of all eligible studies. The minimum reporting checklist (Table 5, Section 8.3) was derived by mapping each recurrent laboratory–clinical gap identified in this synthesis onto a single reportable parameter, informed by existing additive-manufacturing, material, and biological-evaluation standards (ISO/ASTM 52900 [10], ASTM F2924/F3001 [11,12], ISO 5092 [13], and ISO 10993 [14]); it is proposed as a preliminary framework rather than a validated instrument.

A generative AI tool (Claude, a large language model developed by Anthropic, San Francisco, CA, USA) was used during the preparation of this manuscript (2025–2026) to reorganize author-prepared text and improve its linguistic structure. Study selection, evidence weighting, numerical extraction, scientific interpretation, and all conclusions were performed and verified by the authors; full details are given in the Acknowledgments.

### 2.1. Search Strings and Study-Selection Flow

The three core PubMed/MEDLINE strings underlying the search were as follows: String 1 (core material-manufacturing literature): (“porous titanium” OR “Ti-6Al-4V” OR “Ti6Al4V”) AND (“additive manufacturing” OR “3D printing” OR “selective laser melting” OR “electron beam melting”) AND (orthopedic OR orthopaedic OR acetabular OR arthroplasty OR spine OR scaffold) [*n* = 527; rerun 27 August 2026]. String 2 (clinical series for Table 3): (“porous titanium” OR “3D-printed” OR “additively manufactured”) AND (acetabular OR “revision total hip” OR “metaphyseal cone” OR “interbody cage” OR augment) AND (survivorship OR outcomes OR “case series” OR cohort) [*n* = 373]. String 3 (low-modulus alloys): (“beta titanium” OR “low modulus”) AND (alloy) AND (implant OR biomedical OR orthopedic) [*n* = 257; rerun 27 August 2026]. Google Scholar and publisher platforms were used for backward and forward citation chasing on the records these strings retrieved, as described above. Figure 1 summarizes the resulting selection flow for the clinical evidence base: records identified by String 2 and citation chasing were screened at the title/abstract level against the eligibility criteria above; full texts were assessed for the anatomical blocks of Table 3, and 14 studies (12 primary series and 2 pooled analyses) were retained. Because the search was purpose driven rather than exhaustive, Figure 1 documents the provenance of the evidence actually used; it is not an exhaustive enumeration of all eligible literature, and the PRISMA label is deliberately not claimed.

### 2.2. Evidence Grading, Risk-of-Bias Appraisal, and Overlapping Authorship

In response to peer review, the evidence appraisal was formalized in three ways. First, each clinical study in Table 3 was assigned a level of evidence according to the Oxford Centre for Evidence-Based Medicine (OCEBM) 2011 framework [15]; the assignments and their rationale are provided in Table S3 (Supplementary Materials). Under this framework, the single comparative cohort [16] sits at Level III, and the remaining primary series—retrospective, single-arm case series and cohorts—sit at Level IV; the two pooled analyses [17,18] are systematic reviews of Level IV evidence, which the OCEBM framework does not elevate to a higher level, although a systematic review is generally preferable to any individual study within that level [15]. Second, the methodological quality of the twelve primary series was appraised with the MINORS instrument (Methodological Index for Non-Randomized Studies), which was developed and validated precisely for non-comparative and comparative surgical studies and scores eight (non-comparative) or twelve (comparative) items from 0 to 2, with ideal totals of 16 and 24 [19]. Item-level scores are reported in Table S4. The dominant, consistent weaknesses across the series are those inherent to retrospective surgical cohorts: retrospective data collection, absent or unstated blinding of endpoint assessment, incompletely reported attrition, and the universal absence of a prospective sample-size calculation. Third, overlapping authorship in the clinical evidence base is declared explicitly here rather than only among the limitations: the largest fully porous acetabular shell series [2] and two further key cohorts [20,21] share authors and institutions, and the meta-analysis [17] pools cohorts from this same literature. These cohorts were, therefore, treated as related rather than fully independent lines of evidence: they were tabulated separately in Table 3, their figures were not themselves pooled in this review, and its pooled estimates were read as indicative rather than confirmatory, as noted in the caption of Table 3 and in Section 9.

## 3. Why Porous, Why Titanium: Low-Modulus β-Ti Alloys

### 3.1. The Rationale for Titanium in Orthopedics

Titanium and its alloys dominate orthopedic implants because of a set of complementary properties: high specific strength, superior corrosion resistance, low density, and a lower elastic modulus than other metallic biomaterials [22]. Commercially pure titanium (CP-Ti) and Ti-6Al-4V have been the standard materials for bone plates, screws, intramedullary nails, and joint prostheses for decades [1], and this material group has long been called the “ultimate choice” for orthopedic implants [23,24]. Most load-bearing implants in fact use the extra-low-interstitial grade, Ti-6Al-4V ELI (Grade 23), in which reduced oxygen, nitrogen, and hydrogen contents are generally associated with improved ductility and fracture toughness relative to standard Grade 5, although in additively manufactured parts the resulting fatigue behavior is governed as much by microstructure, porosity, and surface condition as by interstitial content—an interstitial-control point that also appears in the reporting framework of Section 8 (Table 5). Measured against the basic requirements, Cui et al. [25] defined for load-bearing biomedical alloys, the limitations of stainless steel, magnesium, and cobalt-based alloys—toxic-element release, high elastic modulus, or rapid degradation—are what make titanium-based alloys stand out for load-bearing orthopedic use. The elastic modulus of these alloys (~103–114 GPa) is lower than that of stainless steel and Co-Cr alloys, but it remains several times that of bone; this is the material-derived core of the stress-shielding problem noted in Section 1.

### 3.2. Low-Modulus β-Ti Alloys: Mechanisms and Limits

Second-generation biomedical titanium alloys were developed to reduce the elastic modulus mismatch at the material level—that is, to bring the implant’s mechanical biocompatibility closer to that of bone [26,27]. In the first stage, α+β-type alloys such as Ti-6Al-7Nb and Ti-5Al-2.5Fe were produced by replacing toxic vanadium with more biocompatible elements such as niobium and iron. The main drop in modulus, however, came with metastable β-type alloys rich in β-stabilizing elements [28]. As Table 1 shows, the elastic modulus of β-type alloys is well below that of α+β alloys: 79–84 GPa for Ti-13Nb-13Zr, 78 GPa for Ti-15Mo, 55–66 GPa for Ti-29Nb-13Ta-4.6Zr (TNTZ), 55 GPa for Ti-35Nb-2Ta-3Zr-0.3O (Gum metal), and 42–46 GPa for Ti-24Nb-4Zr-7.9Sn (Ti-2448) [1]. Several of these alloys trace back to landmark studies. The Gum metal family was introduced as a class of multifunctional alloys that, when cold-worked, combine ultra-low elastic modulus, ultra-high strength, and superelasticity through a dislocation-free deformation mechanism [29].

The term denotes a group of multifunctional β-titanium alloys based on the Ti-Nb-Ta-Zr-O system, whose compositions are selected to satisfy three simultaneous electronic and structural criteria—a compositional average valence electron concentration, a bond order, and a d-electron orbital energy level—with Ti-23Nb-0.7Ta-2Zr-1.2O and Ti-35Nb-2Ta-3Zr-0.3O as representative members [29].

A consideration specific to low-modulus implants should be noted at this point, because it recurs for porous architectures in Section 3.3. Lowering component stiffness increases load transfer to the surrounding bone, but a more compliant implant also deforms more under physiological load, which can increase relative micromotion at the bone–implant interface in the early postoperative period—precisely when initial stability determines whether bone ingrowth or fibrous encapsulation follows. Reduced stiffness is, therefore, not a monotonic benefit: it must be balanced against the primary stability that osseointegration requires, and this trade-off applies equally to low-modulus alloys and to highly porous architectures.

Likewise, Ti-24Nb-4Zr-7.9Sn (Ti-2448) was characterized for biomedical use by its low modulus and superelastic behavior [30]. Such low-modulus alloys can also be reached by other manufacturing routes: processing Ti2448 by powder metallurgy, Guo et al. [31] obtained an elastic modulus of ~44 GPa, a compressive yield strength of 1100 MPa, and a fracture elongation of 17.5% after solution treatment and quenching. Even this figure sits well above cortical bone, which shows that the practical floor for second-generation β-Ti alloys in bulk form cannot reach the bone modulus—and that the very low effective modulus sought clinically comes mainly from porous architecture.

**Table 1:** Representative elastic modulus values of selected biomedical titanium alloys and bone. Values are drawn predominantly from the review by Gao et al. [1]; certain alloys are anchored to landmark primary studies in the field: Ti-24Nb-4Zr-7.9Sn/Ti-2448 (Hao et al. [30]), Gum metal/Ti-Nb-Ta-Zr-O (Saito et al. [29]), and the metastable β-type alloy line, including Ti-29Nb-13Ta-4.6Zr (TNTZ) and Ti-13Nb-13Zr, whose low moduli (~55–84 GPa) were established in the foundational β-Ti design and characterization work of Kuroda et al. [28] and Niinomi [26,27]. Reported values are representative literature figures and may vary with processing condition (annealing, cold rolling, and porosity) rather than independently pooled measurements. † The unusually wide range for TLM (45–81 GPa) reflects its strong dependence on thermomechanical processing and retained β-phase fraction rather than a single characteristic value. The Ti-12Mo-6Zr-2Fe (TMZF) modulus is as reported in the hip arthroplasty literature [32] rather than in [1]; the clinical status column reflects the translation record discussed at the end of Section 3.2.

Structure	Material	Elastic Modulus (GPa)	Clinical Translation Status (Representative Implant)
—	Cortical bone	7–30	—
—	Cancellous bone	0.3–4	—
α	CP-Ti	103–107	Established clinical use (plates, screws, joint components)
α+β	Ti-6Al-4V	110–114	Clinical standard for load-bearing implants; substrate of the AM porous implants reviewed here
α+β	Ti-6Al-7Nb	110	Translated; Zweymüller-type femoral stems [33]
β	Ti-13Nb-13Zr	79–84	Standardized (ASTM F1713 [34]); introduced by Smith & Nephew in the 1990s; no widespread current load-bearing use identified [35]
β	Ti-15Mo	78	Standardized (ASTM F2066) [36]; no commercial load-bearing orthopedic implant identified
near-β	Ti-12Mo-6Zr-2Fe (TMZF)	~70 [32]	Translated (Accolade femoral stem); exited after head-neck taper corrosion with paired CoCr heads [37,38]
β	Ti-29Nb-13Ta-4.6Zr (TNTZ)	55–66	Laboratory/preclinical [39]
β	Ti-35Nb-2Ta-3Zr-0.3O (Gum metal)	55	Laboratory/preclinical in load-bearing orthopedics [29]
β	Ti-24Nb-4Zr-7.9Sn (Ti-2448)	42–46	Laboratory; AM porous scaffolds at research stage [1]
β	Ti-3Zr-2Sn-3Mo-25Nb (TLM)	45–81 †	Laboratory [1]

The metallurgical mechanisms behind the low modulus follow directly: the modulus shows a “W-shaped” dependence on β-stabilizer content and mean valence-electron/atom (e/a) ratio, with phase moduli ordered β ≈ α″ < α′ < α < ω [1]. A low-modulus design, therefore, aims to retain the metastable β phase while controlling the formation of the hardening α″ martensite and the ω phase; multi-element systems such as Ti-3Zr-2Sn-3Mo-25Nb (TLM) illustrate the breadth of this design space, spanning 45–81 GPa with processing. Yang et al. [40] offer a direct illustration: raising β-phase stability through Zr additions in d-electron-designed Ti-25Nb-2Mo-xZr alloys suppressed the high-modulus ω phase, lowering the modulus to 56.3 GPa while raising elongation at fracture to 48.2%. Thermomechanical treatment pushes the balance further—high-reduction cold rolling reaches ~42 GPa in Ti-35Nb-4Sn and preserves ~39–41 GPa in low-β-stability alloys while increasing strength [1]—and TRIP/TWIP effects together with oxygen addition allow favorable strength–ductility balances; these composition and processing effects have been reviewed comprehensively [41]. As an extreme case, Saito et al. [29] introduced the Ti-Nb-Ta-Zr-O-based Gum metal alloys, which combine low elastic modulus, high strength, and superelasticity through an unusual “dislocation-free” deformation mechanism.

Even so, the elastic modulus of bulk β-Ti alloys cannot easily be pushed below a certain floor. The lowest bulk values still hover around 40 GPa (~39–41 GPa in the cold-rolled, low-β-stability alloys above)—above the upper limit of cortical bone (~30 GPa)—and the requirement to use non-toxic elements raises fresh biocompatibility questions in multi-element, high-concentration designs. On this point, Rabbitt et al. [42] argue that current biomedical titanium alloys are mostly borrowed from other industries and that this contributes to biologically driven failure; they call for a shift from the simple “biocompatible and mechanically stable” model toward designs that account for biological responses—immune response, angiogenesis, osseointegration, and infection—from the outset. There is also an intrinsic tension in metastable β-Ti alloys between low modulus and strength: as the modulus falls, strength tends to fall with it, which limits fatigue resistance and raises the risk of implant failure in clinical use [43]. On top of this, producing low-modulus metastable β-Ti alloys demands complex metallurgy and carries a high final cost, further constraining their commercial and clinical use [44]. Achieving full mechanical compatibility with bone through alloying alone is, thus, practically limited.

Which of these alloys have actually crossed into clinical hardware is itself informative, and the record is short. The vanadium-free substitution that translated most widely is not a low-modulus β alloy at all but α+β Ti-6Al-7Nb, used in clinically established rectangular tapered femoral stems of the Zweymüller type [33]. Among genuinely low-modulus metastable β alloys, the most instructive case is Ti-12Mo-6Zr-2Fe (TMZF), with an elastic modulus of about 70 GPa reported in the arthroplasty literature [32]: it reached large-scale clinical use in a commercial femoral stem, and its clinical exit appears to have been unrelated to its modulus advantage. Retrieval series documented mechanically assisted fretting-crevice corrosion and gross trunnion failure at the head-neck taper when these stems were paired with particular cobalt–chromium femoral heads [37,38]; the implicated heads were recalled in 2016 [37], and the alloy was not carried forward in the successor stem design [45], which is reported to use conventional Ti-6Al-4V [46]—although satisfactory mid-term survivorship of the TMZF stem itself has also been reported [47]. The remaining low-modulus alloys of Table 1 have not followed even that path. Ti-13Nb-13Zr was introduced by Smith & Nephew in the 1990s [35] and is specified in ASTM F1713 [34], and Ti-15Mo is likewise covered by a dedicated implant-alloy standard, ASTM F2066 [36]; but we could not identify a widely used commercial load-bearing orthopedic implant manufactured from either alloy, and TNTZ, Gum metal, Ti-2448, and TLM remain at the laboratory and preclinical stages in load-bearing orthopedics [39]. Two conclusions follow. First, clinical translation of an implant alloy is decided at the level of system behavior—fatigue, wear, junction tribocorrosion, manufacturability, and regulatory precedent—rather than by the single property the alloy was designed to optimize; the TMZF experience, in which a low-modulus alloy succeeded as a material and failed at a modular junction, is the concrete case, and it connects directly to the taper-corrosion considerations raised in Section 1. Second, this record is summarized in the clinical-status column of Table 1: the only low-modulus route with broad current clinical presence is the architectural one that the remainder of this review examines.

### 3.3. Porous Architecture: Lowering the Effective Modulus Through Architecture

Porous titanium alloys offer an alternative—and currently the more clinically established—way to overcome this limit. In a porous structure, elastic modulus and compressive strength both fall as porosity rises, so by tuning pore size, shape, and distribution the effective modulus can be matched to the target bone tissue [1]. The range this opens up is far lower than anything reachable in bulk alloys. As reviewed by Gao et al. [1], SLM- and EBM-produced porous Ti-24Nb-4Zr-7.9Sn (Ti-2448) scaffolds reached elastic moduli of 0.95 GPa and 1.34 GPa, respectively, while porous Ti-35Nb-2Ta-3Zr can reach ~3.1 GPa—close to cancellous bone—at larger pore sizes. With the pore size held in the 500–800 µm range, the modulus can, instead, be tuned to ~15–20 GPa, close to that of the femur. Pore size, however, is only one half of the architectural description. The same effective modulus depends equally on the dimensions of the struts that bound those pores, and the strut dimensions that can actually be realized are set by the manufacturing process rather than chosen freely by the designer. This manufacturing constraint is taken up in Section 4.3.

**Figure 2:**
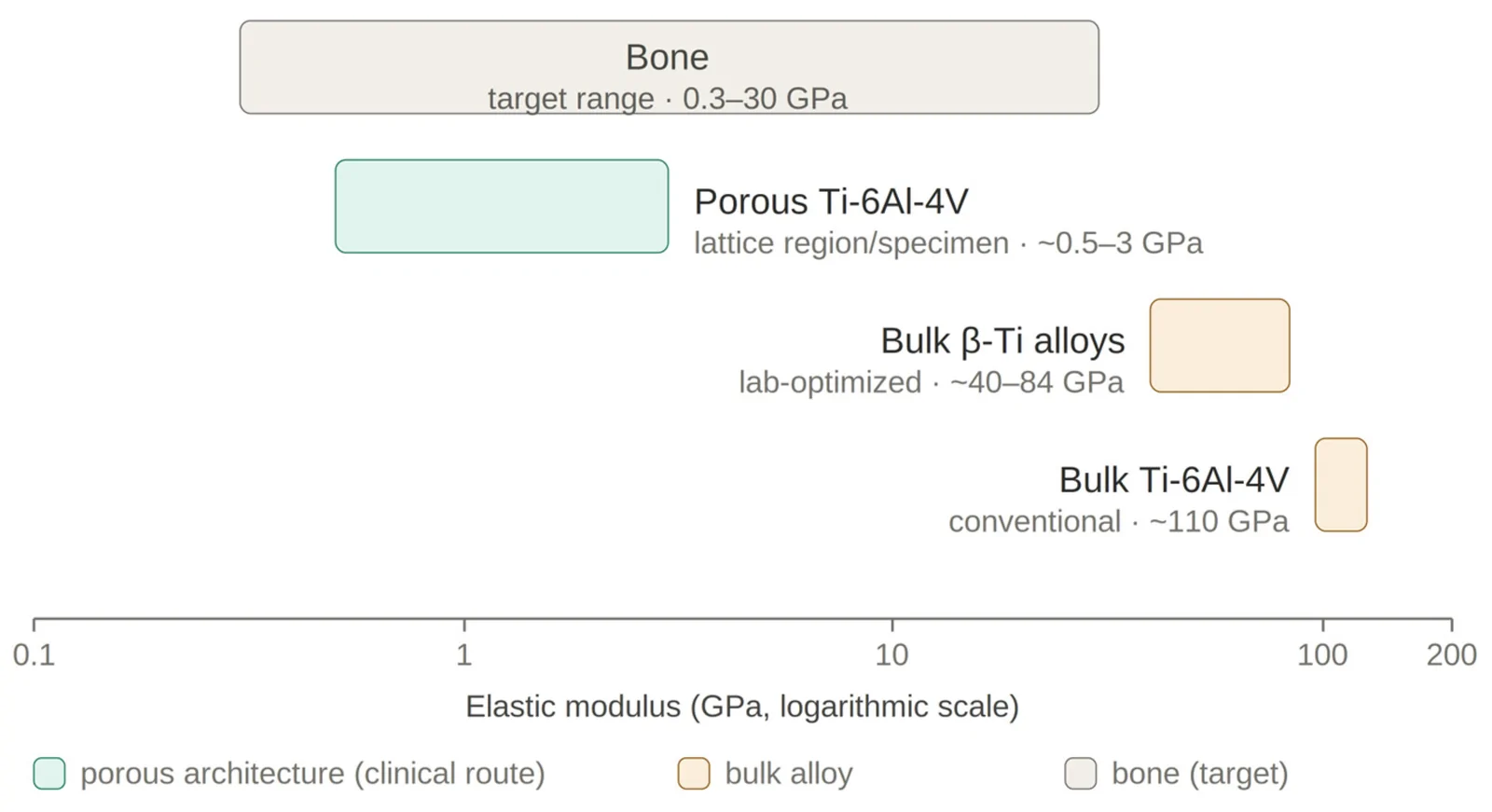
Elastic modulus scale for load-bearing orthopedic implants (logarithmic axis). The effective modulus reported for porous Ti-6Al-4V lattice regions/specimens (~0.5–3 GPa) descends into the bone target range (0.3–30 GPa), whereas the low-modulus bulk β-Ti alloys developed in the laboratory (~40–84 GPa) and conventional bulk Ti-6Al-4V (~110 GPa) remain at higher modulus. This value is the effective structural modulus of the porous architecture—a coupon- or lattice-region property—not the overall stiffness of a whole implant component, which typically combines a solid shell with a porous region. The key point is that the low effective modulus is obtained through porous architecture rather than through alloying.

Here the review’s core argument takes concrete form. In the clinically most critical settings—above all acetabular reconstruction—the low effective modulus comes not from low-modulus β-Ti alloys but from the porous architecture of conventional Ti-6Al-4V; this architectural route is what, in practice, bridges the alloy-level achievements of materials science and today’s clinical solutions (Figure 2). This is not to dismiss β-Ti alloys but to identify the route that current clinical translation has mostly taken; they remain important translational candidates as questions of fatigue, manufacturability, cost, and regulatory approval are resolved. This framework is the logical backbone of the sections that follow (Section 4, Section 5, Section 6 and Section 7).

Porous metal fixation surfaces are not new to orthopedics. Among the earliest highly porous constructs to enter clinical use was Hedrocel, a chemical-vapor-deposition tantalum structure marketed as Trabecular Metal, which established that an open, bone-like porous architecture could achieve durable biological fixation well before additive manufacturing entered the field [48]. The additively manufactured Ti-6Al-4V architectures reviewed here are, therefore, a manufacturing development within an established fixation concept rather than a departure from it—a point that bears directly on the evidentiary bar these devices should meet, taken up in Section 7.

## 4. Advanced Manufacturing: Additive Manufacturing (AM)

The central argument of Section 3 points directly to manufacturing technology. Conventional powder-metallurgy methods can produce porous titanium, for instance, interconnected pores of ~100–500 µm suitable for osseointegration together with a compressive strength of 50–300 MPa. In these methods, however, control over the pore architecture is limited and mostly stochastic. The technology that can precisely produce complex, patient-specific, deterministic porous internal structures is additive manufacturing (AM) [1,49].

### 4.1. Additive Manufacturing Methods

By forming process, metallic AM technologies fall into methods such as selective laser melting (SLM; ISO/ASTM designation PBF-LB/M), directed energy deposition (DED), selective laser sintering (SLS), electron beam melting (EBM; ISO/ASTM designation PBF-EB/M), laser cladding (LC), and wire-arc additive manufacturing (WAAM). For consistency, this review uses “SLM” and “EBM” throughout. Of these, SLM and EBM are the most widely used for medical titanium alloys because of their relatively high precision, and the Ti-6Al-4V-based implants distributed clinically are reported to come mainly from EBM and SLM platforms [1,3].

In SLM, a laser beam selectively melts pre-spread metal powder to build the part layer by layer, working in an inert-gas environment along a predetermined scan path. Because unused powder can be recovered, the method is highly material efficient. Its relatively high forming accuracy suits it to small, high-precision components; the trade-offs are a low build rate, high residual stress in the finished part (which calls for stress-relief heat treatment), and—depending on the part geometry—a need for support structures to anchor the part, manage overhangs, and control distortion. EBM, instead, uses a high-energy electron beam, steered by an electromagnetic deflection coil, as the heat source. Its dimensional accuracy is lower but its forming efficiency higher, and because the process chamber is held at around 650–700 °C, the residual stresses are lower than in SLM, so a dedicated stress-relief step is often unnecessary—although HIP or other thermal post-processing may still be applied to meet density, fatigue, microstructure, or component-qualification requirements [1].

The difference in the solidification rate between the two methods has a decisive effect on the microstructure. The high cooling rates in SLM (up to ~10^6^ K/s) favor acicular α′ martensite, whereas the slower cooling in EBM produces a lamellar α or (α+β) basketweave structure—a distinction detailed in Section 5. The heat-source spot size also affects the surface quality: the electron-beam spot (~200 µm) is much larger than the laser spot (~40 µm), so EBM creates a wider melt pool and a rougher surface, while SLM specimens are smoother [1]. Comparing EBM- and SLM-produced Ti-6Al-4V with forged and cast material, Murr et al. [50] characterized the α (hcp), β (bcc), and α′ martensite phases by optical and electron microscopy and showed early on that these methods can produce prostheses and implants directly from CT/MR data.

### 4.2. Defects in Additively Manufactured Titanium

Because AM involves rapid heating, cooling, and solidification, it is prone to microstructural defects—lack of fusion, porosity, inclusions, and microcracks—that can cause early structural failure if left uncontrolled. Defect formation depends heavily on process parameters such as scan speed, laser power, scan strategy, hatch spacing, and powder-layer thickness. As compiled in the review by Gao et al. [1], very high scan speeds (reported there as >1300 mm/s) tend to produce lack-of-fusion defects, while very low speeds (<550 mm/s) tend to produce keyhole defects; the specific thresholds are process- and machine-dependent, but the underlying direction—too little energy leaves unmelted lack-of-fusion pores, too much energy drives keyhole porosity—is well established in primary process-window studies of SLM Ti-6Al-4V [51]. The mechanical cost of these defects has also been demonstrated experimentally. In SLM-produced Ti-6Al-4V, Ramirez et al. [51] found that varying keyhole and lack-of-fusion porosity and surface roughness (Sa ≈ 15 → 7 µm) shifted fatigue life by about 10^2^ cycles, and that the Basquin stress–life relationship fit the process conditions well except under keyhole porosity. What matters is not only whether a defect is present but its size and location. Mapping every pore before testing by X-ray computed tomography in EBM-produced Ti-6Al-4V, Tammas-Williams et al. [52] showed that without HIP, fatigue life was dominated by porosity and scattered widely, with crack initiation accounting for more than 70% of the life. Strikingly, the defect that starts the crack is often not the largest one; once proximity to the free surface and pore aspect ratio are accounted for, the actual initiator was found to fall within the most harmful 3%.

Pore defects are common in AM. Gas pores are usually smaller than ~100 µm and roughly spherical, arising mainly from the following three sources: gas inclusions within the powder; gas entrapment from poor flowability during powder spreading or feeding; and the keyhole mechanism, in which the laser’s high energy density rapidly vaporizes metal. Lack-of-fusion defects, by contrast, are typically larger, irregular, and sharp-cornered and are correspondingly more damaging to fatigue. The main strategies for reducing porosity are choosing powders with high sphericity and flowability, optimizing process parameters, and controlling the shielding-gas volume [1]. The manufacturing method also shapes the surface: da Costa Valente et al. [53] found that SLM-produced Ti-6Al-4V discs carried partially melted spherical particles while subtractively machined discs showed a grooved topography, with the two methods differing sharply in wettability, topography, and surface chemistry. A concern specific to porous rather than solid parts arises at this point. An open-cell lattice is built inside a bed of loose powder, and in EBM the surrounding powder is additionally held in a lightly sintered cake by the elevated chamber temperature; unmelted and partially sintered powder is, therefore, trapped throughout the internal channels of the part when the build ends. Removal is a process step in its own right, not an incidental one, and its difficulty scales with the tortuosity of the architecture and inversely with pore size—precisely the direction in which designs move when finer struts and smaller pores are used. The routes in routine use are mechanical—for EBM, powder-recovery blasting of the sintered powder cake with powder of the same alloy—followed by chemical and electrochemical treatments—acid pickling, chemical polishing, or electropolishing—that additionally dissolve the partially melted particles fused to the strut surface, with ultrasonic cleaning in the final stage [54,55]. These steps are not mechanically neutral: chemical and electrochemical removal thins the struts and so alters relative density and effective modulus, but by removing surface notches and adherent particles, it can raise the fatigue strength, so the trade-off must be characterized rather than assumed [56]. Verification is the weaker link. Visual inspection cannot establish that the internal channels of a lattice are clear; micro-computed tomography, gravimetric checks, and validated cleaning protocols with residue, bioburden, and endotoxin testing are required, and the difficulty of validating cleaning for complex internal geometries is explicitly recognized in regulatory guidance for additively manufactured devices [57]. This is also where the manufacturing and clinical arguments of this review meet: the particles that survive these steps are the same particles implicated in third-body wear, raised bacterial adhesion, and suppressed osteogenic activity in Section 7.5. Post-processing and cleaning status should, accordingly, be reported as a manufacturing parameter (Table 5), which clinical series at present almost never do. Those partially melted particles are also the source of the debris and third-body wear risk addressed in Section 7.5.

### 4.3. Porous Architecture Design

The mechanical behavior of porous structures is closely tied to unit-cell characteristics. As a rule, compressive strength and elastic modulus fall as porosity rises, so tuning pore size, shape, and distribution lets the effective modulus be matched to the target bone. That porous architecture lowers the effective modulus and thereby reduces stress shielding is a core principle also stressed in independent reviews [9,58], and there is direct experimental support for it. Optimizing the micro-architecture of a fully porous 3D-printed titanium femoral stem to mimic the local bone, Arabnejad et al. [59] showed in composite femurs—using semi-physiological experiments and a finite-element model—that stress-shielding-related bone loss could be cut by 75% relative to a fully solid implant. Finite-element analyses show that pore geometry strongly controls the modulus; honeycomb pores, for example, produced the highest and square pores the lowest elastic modulus. With the pore size held in the 500–800 µm range, an elastic modulus of 15–20 GPa—close to the human femur—can be obtained [1].

Achievable strut dimensions deserve the same attention as pore size, since for a given unit cell the strut diameter—together with the pore size—fixes the relative density and, therefore, the effective modulus and strength of the architecture. Strut dimensions are not, however, a free design variable. The smallest strut that can be formed reliably is bounded from below by the heat-source spot size, the layer thickness, and the particle size distribution of the feedstock: a strut cannot be built appreciably narrower than the melt pool that forms it, and dimensional fidelity degrades as the strut diameter approaches the size of the individual powder particles. The consequences differ between the two dominant platforms. As noted in Section 4.1, the electron-beam spot (~200 µm) is substantially larger than the laser spot (~40 µm) [1], and EBM is operated with a coarser feedstock than SLM [60,61]; EBM, therefore, has a coarser minimum feature size and a rougher as-built strut surface, whereas SLM can resolve finer struts. Powder morphology acts through the same chain. High sphericity and good flowability are already identified in Section 4.2 as the principal means of limiting pore defects [1]; irregular or satellited particles impair layer spreading and packing, and repeated powder reuse progressively degrades morphology and raises interstitial oxygen content [60,61]. Two dimensional consequences follow and are relevant to every mechanical value quoted for a lattice. First, partially melted particles adhere to the strut surface, so the as-built strut is thicker than designed while its effective load-bearing cross-section is smaller—the as-designed and micro-CT-measured strut diameters routinely differ [62,63], a deviation that weighs proportionally more heavily on thinner struts. Second, because surface roughness is largely independent of strut diameter, its relative severity—and, hence, the notch effect discussed in Section 5.4—increases as the struts are made thinner. Designed strut dimensions should, therefore, always be reported alongside the micro-CT-measured values, as required in the framework in Table 5.

Porous architecture can be built as uniform structures or as gradient/functionally graded material (FGM) structures, in which porosity or cell type varies with location. Two unit-cell families stand out: strut-based lattices (e.g., diamond) and triply periodic minimal surface (TPMS)-based structures (e.g., gyroid). Their advantages are complementary—strut-dominated structures such as diamonds offer higher stiffness and strength, while TPMS structures such as gyroids favor cell/bone ingrowth and fatigue behavior through higher permeability and more uniform stress distribution. Characterizing additively manufactured Ti-6Al-4V gyroid and diamond sheet-TPMS structures experimentally and by finite-element methods, Naghavi et al. [64] found that within a suitable porosity range both reached the cortical-bone range in stiffness and yield strength (~6–30 GPa and ~125–210 MPa) and that at the same pore size the diamond structure was about 65% stiffer and 48% stronger in compression than the gyroid. TPMS structures can also mimic several bone properties at once: Bobbert et al. [65] showed that 16 porous Ti-6Al-4V biomaterials based on four TPMS types produced by SLM reproduced the topology of trabecular bone (mean curvature near zero), pairing a low elastic modulus—enough to avoid stress shielding—with a strength high enough for mechanical support, while also achieving exceptionally high fatigue resistance and a permeability within the range reported for bone. Mechanistically, these geometric differences govern not just the effective modulus but also mass transport and the cellular response: high interconnected permeability eases nutrient and waste diffusion and vascularization, supporting bone ingrowth deep into the scaffold, while the continuous, near-zero-mean-curvature TPMS surface tends to offer a better substrate for osteoblast attachment and spreading than strut–node lattices. Unit-cell selection (e.g., diamond–gyroid) is, therefore, not a purely mechanical choice but a simultaneous trade-off between mechanical goals (modulus, strength, and fatigue) and biological ones (permeability and ingrowth). For bone ingrowth, specifically, a pore size of ~300–600 µm is commonly favored [66] (developed with in vivo evidence in Section 6.1). The node-free, continuous TPMS surface also reduces stress concentration and so improves fatigue behavior (Section 5).

The ranges quoted in this section should be read with an explicit qualification. The windows commonly cited—approximately 300–600 µm for bone ingrowth [66] and 500–800 µm with a corresponding effective modulus of 15–20 GPa for modulus matching [1]—are drawn from heterogeneous sources: small-animal ingrowth models, coupon-level mechanical testing, and finite-element analyses, each with its own unit cell, specimen geometry, loading mode, and endpoint. They are not the product of comparative evaluation within a single load-bearing application, and they should not be treated as a single optimum transferable across applications. The mechanical demands, in fact, differ substantially from one indication to another. An acetabular shell transmits predominantly compressive loads through a solid hemispherical substrate carrying a comparatively thin porous interface layer; a lumbar or cervical interbody cage must resist subsidence into the vertebral endplate while accommodating a graft window; a metaphyseal cone or sleeve carries compressive, shear and torsional loads in deficient cancellous bone; an acetabular augment is loaded through screws over a small contact area; and a segmental tumor prosthesis must carry bending and torsion over a long span. The biological target differs in parallel, since the host bed may be cortical, cancellous, sclerotic, irradiated, or previously infected. A porosity that is appropriate for a thin interface layer supported by a solid shell is, therefore, not necessarily appropriate for a fully porous cone or a segmental replacement, and the fatigue consequences of a given porosity depend on the loading regime of the specific component, as set out in Section 5.4. On the evidence available to date, a single optimal porosity or pore-size range for load-bearing porous titanium implants, in general, does not appear to be established. What the evidence does support is a design envelope—derived largely from preclinical ingrowth models and coupon mechanics—within which application-specific optimization must still be carried out. In our search, we did not identify a clinical study comparing different pore sizes or porosities head-to-head within the same application against a clinical endpoint, and this absence is itself an instance of the laboratory–clinical gap that the review is built around. Porosity ranges should, accordingly, be reported and interpreted as application-specific (Table 4, “Pore size/porosity” row), not as a universal specification.

### 4.4. Commercial AM Implants in Clinical Use

The clinical maturity of additive manufacturing is best seen in the products now on the market. The Delta TT porous titanium acetabular system (Lima Corporate, Villanova di San Daniele del Friuli, Italy, now part of Enovis, Dallas, TX, USA; EBM-produced Trabecular Titanium) is CE-marked and reached the U.S. market through the FDA 510(k) pathway (K112898; multi-hole variant K200656) rather than premarket approval, and DePuy Synthes’ (Raynham, MA, USA) 3D-printed CONDUIT interbody portfolio—whose cages have an elastic modulus close to trabecular bone—is built on EIT Cellular Titanium, likewise 510(k)-cleared (K170503). Further examples compiled by Gao et al. [1], whose regulatory pathways are not independently verified here, include Joimax’s NEST fusion device (Karlsruhe, Germany; porous core with solid edges, modulus ~3 GPa), an AM porous acetabular cup marketed by Beijing Aikang Medical (Beijing, China) in 2015, and porous titanium hip, knee, and spinal products from Double Medical (Xiamen, China). Porous titanium AM implants have, in short, moved beyond the laboratory into regulatory-cleared clinical products.

Still, almost all of these commercial products—despite the progress in low-modulus β-Ti alloys—are based on conventional Ti-6Al-4V. The microstructure and fatigue behavior of these additively manufactured structures are the fundamental determinants of clinical durability, and they are the subject of the next section.

## 5. Microstructure and Fatigue Behavior

The manufacturing parameters of Section 4 directly set the microstructure and, with it, the mechanical performance. For a load-bearing implant, the most critical property is not static strength but fatigue behavior under cyclic loading, since the implant endures millions of load cycles over its lifetime. This section covers the microstructural development of additively manufactured titanium, the link between phase stability and elastic modulus, the role of heat treatment, and, finally, the fatigue behavior that ultimately governs clinical durability.

### 5.1. Microstructural Development

Because AM solidifies rapidly, grains tend to grow epitaxially along the build direction, often yielding a columnar grain structure and a non-uniform, anisotropic microstructure—undesirable for biomedical use. In the review by Gao et al. [1], the scan strategy strongly influences this: cross-scanning can refine columnar grains, resulting in a more uniform microstructure and higher hardness. Raising the scan speed shortens the laser–powder interaction time, shrinks the melt pool, and increases the cooling rate, producing finer grains, while raising the laser energy density improves the melt-pool overlap and so reduces defects. These process effects have also been shown in porous structures: evaluating five build orientations and three heat-treatment conditions in SLM-produced Ti-6Al-4V diamond lattices, Wauthle et al. [67] found that both build direction and heat treatment are decisive for the microstructure and static mechanical properties of the lattices—so values from bulk specimens cannot simply be transferred to lattice struts.

The manufacturing method also decisively shapes the phase composition. SLM-produced Ti-6Al-4V is made up mainly of acicular α′ martensite because of the high cooling rate (~10^6^ K/s), whereas EBM-produced material shows a lamellar α or (α+β) basketweave structure because the process chamber is held at around 650–700 °C [1,67].

### 5.2. Phase Stability and Elastic Modulus

As introduced in Section 3.2, the phase elastic moduli follow the order β ≈ α″ < α′ < α < ω, and the β-Ti modulus varies in a “W-shaped” pattern with β-stabilizer content and a mean e/a ratio. For Ti-6Al-4V, specifically, Gao et al. [1] report that as the α-phase volume fraction rises, the modulus first falls, bottoms out at about 50% α, and then climbs again—so tuning the α-phase content through heat treatment can optimize strength and modulus together.

SLM-produced Ti-6Al-4V typically shows low ductility and marked mechanical anisotropy because of its acicular α′ martensite and columnar prior-β grains. Post-processing heat treatment is, therefore, commonly used to decompose the α′ martensite into balanced α+β phases. By adjusting the SLM process variables (layer thickness, energy density, and focal offset), Xu et al. [68] decomposed the acicular α′ martensite in situ into an ultrafine (~200–300 nm) lamellar α+β structure; keeping the yield strength above 1100 MPa, they raised the total elongation to 11.4%, outperforming both conventional SLM and mill-annealed Ti-6Al-4V.

### 5.3. Post-Processing Heat Treatment

Because AM subjects the material to steep temperature gradients, rapid cooling and thermal-stress buildup can create chemical heterogeneity and unstable microstructures that impair plasticity and lower fatigue strength. Post-processing heat treatment is decisive in addressing these problems. Reviewing post-processing methods for 3D-printed titanium implants in terms of mechanical, osseointegrative, and antibacterial improvement, Li and Wang [69] stress that post-processing is indispensable for achieving the desired implant performance. Two lines of evidence describe the same underlying transformation. In the compilation of Gao et al. [1], annealing Ti-6Al-4V at 850 °C raised elongation from 7.3% to 12.8%, with progressively higher annealing temperatures transforming acicular α′ martensite into lamellar α+β. The primary experimental basis for this behavior is the work of Vrancken et al. [70], who showed that heat-treatment optimization for SLM-produced Ti-6Al-4V differs fundamentally from that for conventionally processed material: annealing at 1123 K (~850 °C) for 2 h followed by furnace cooling converts the α′ martensite into a lamellar α+β mixture and raises elongation at fracture from ~7.4% in the as-built condition to ~12.8%. Across the temperature series examined (850, 950 and 1050 °C), elongation increased and yield strength fell while the elastic modulus changed negligibly—a point of practical importance here, since heat treatment recovers ductility without altering the modulus mismatch that porous architecture is intended to address.

Heat treatment improves fatigue and corrosion performance mainly by relieving residual stress and homogenizing the microstructure; closure of internal pores, by contrast, requires hot isostatic pressing (HIP) rather than heat treatment alone. Above 600 °C in an insufficiently protected, oxygen-containing atmosphere, a brittle oxide layer readily forms on titanium and degrades both mechanical properties and surface integrity, so vacuum or protective-atmosphere processing becomes necessary—adding complexity and cost [1]. Li and Affolter [71] argue that HIP is the single most effective post-process for the fatigue performance of SLM Ti-6Al-4V, since it largely eliminates internal porosity and reduces residual stress while homogenizing the microstructure. Xi et al. [72] found that standard heat treatment and HIP produce similar tensile strength with no significant difference in low-cycle fatigue, and Jamshidi et al. [73] stress that post-process design must balance mechanical strength against biological performance.

### 5.4. Fatigue Behavior: The Bridge Between Microstructure and Clinical Fracture

Because orthopedic implants face cyclic stresses from the internal environment, a defined fatigue performance is essential for reliability and longevity. Under the ASTM F382-17 standard for metallic bone plates [74], fatigue performance is typically evaluated against a run-out criterion, commonly on the order of one million cycles without failure [1]. But that standard is specific to bone plates: there is no directly equivalent, agreed-upon fatigue protocol for porous acetabular components, cages, and augments—a gap addressed in Section 8.

Component-level standards do exist for the two implant classes most relevant to this review. ISO 7206-4 specifies the determination of endurance properties and performance of stemmed femoral components under cyclic loading, and ASTM F3090 provides a standard test method for the fatigue testing of acetabular devices for total hip replacement; both are routinely applied by testing laboratories during regulatory evaluation [75,76]. What these standards specify, however, is the fatigue performance of the finished component under defined worst-case loading. They do not prescribe how the porous region itself is to be characterized—its as-built strut geometry, its defect population, or its surface condition—and it is at that level that the laboratory fatigue data reviewed below actually vary. The gap is, therefore, not an absence of component-level standards but an absence of agreed reporting at the architectural level, which is the subject of Section 8.

A terminological distinction is needed before the fatigue evidence is read, because the word “porosity” is used in this literature for two physically different things. The first is the designed architectural porosity of the lattice: intentional, deterministic, open and interconnected, on the order of hundreds of micrometers, and the feature that defines the load paths and sets the effective modulus. The second is the process-induced defect population inside the struts themselves: unintended, stochastic, typically tens of micrometers, and comprising the lack-of-fusion, keyhole and gas pores described in Section 4.2. The two act on different length scales and through different mechanisms. Architectural porosity governs the homogenized properties of the structure—it lowers the effective modulus and strength by reducing the load-bearing cross-section and by placing the strut ligaments in bending and tension, with the local stress peaking at the nodes, as Lietaert et al. [77] showed. Intra-strut defects do not appreciably change the effective modulus; they act as local stress concentrators within the strut ligament and determine where and when a fatigue crack starts. Three findings already cited make the second mechanism concrete. Tammas-Williams et al. [52] showed that the initiating defect is often not the largest one, but the one whose proximity to the free surface and aspect ratio place it in the most harmful 3%. Li and Affolter [71] reported that fatigue cracks in additively manufactured Ti-6Al-4V originate mostly from lack-of-fusion and gas pores, that 85–90% of internal defects lie within 200 µm of a free surface and that the irregular, notch-like geometry of lack-of-fusion defects makes them more harmful than spherical gas pores. Masuo et al. [78] showed that removing near-surface pores by hot isostatic pressing raised fatigue strength markedly, while the surface roughness continued to exert a strong negative effect. The interaction between the two populations is what matters for lattices specifically. A defect of a given size that is inconsequential in a bulk coupon may occupy an appreciable fraction of the cross-section of a strut a few hundred micrometers across, and the same applies to surface notches and adherent partially melted particles, which act as external defects whose depth relative to the strut diameter grows as the struts are made thinner (Section 4.3). The defect tolerance, therefore, falls as the strut size falls, and hot isostatic pressing—which closes internal pores but cannot remove surface notches—addresses only one of the two. Read this way, the fatigue penalty of the porous structures documented below is not a penalty of the designed architecture as such: the architecture sets the stress state, and the defect and surface population determine whether the strut survives it. This is why the relative density and designed architecture, on the one hand, and the defect quantity, surface roughness metrics and post-processing status, on the other, are listed as separate reporting items in Table 5, rather than being merged under a single porosity figure.

**Figure 3:**
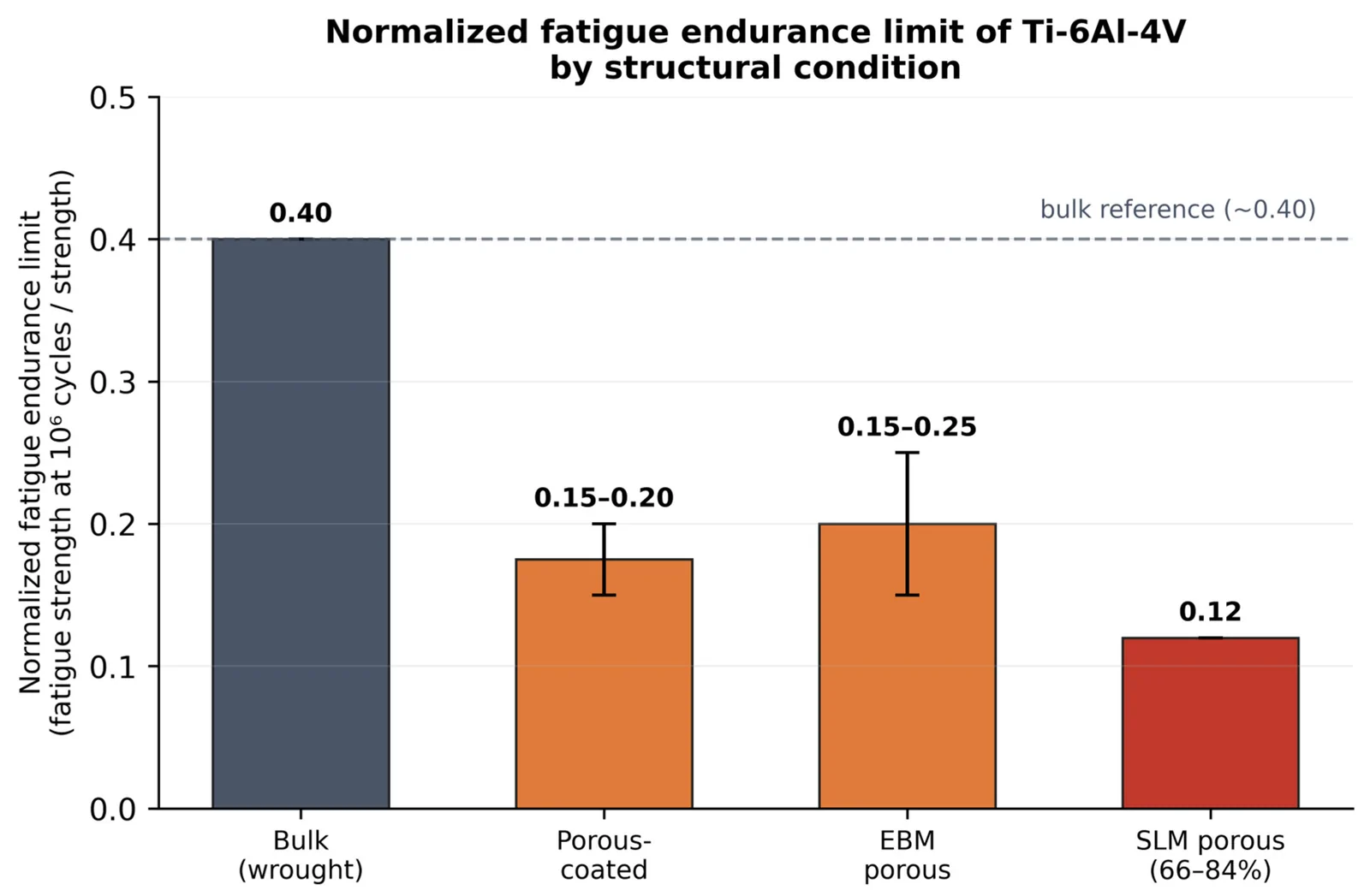
Normalized fatigue endurance limit (fatigue strength at 10^6^ cycles divided by the material’s yield or ultimate strength, as reported by each source) of Ti-6Al-4V across structural conditions, compiled from the verified values in Table 2. Conventional porous architectures fall well below bulk material. The whiskers on the porous-coated and electron beam melting (EBM)-porous bars span the reported literature range for those conditions (0.15–0.20 and 0.15–0.25, respectively); they denote the spread of reported values across heterogeneous studies—differing in porosity, R ratio, loading mode, surface condition, and normalization basis—not a statistical interval (confidence interval, standard deviation, or standard error). The bars are, therefore, indicative rather than directly comparable point for point. A variable-diameter dense-core design has separately been reported to recover a near-bulk value (~0.5), but as this rests on a single figure compiled in [1] and awaiting independent confirmation, it is discussed in the text rather than plotted here. Original figure; values as reported in the cited sources.

The fundamental trade-off of the porosity now becomes clear: a porous structure eases stress shielding by lowering the elastic modulus, but it also lowers the fatigue resistance. This trade-off is well documented. Amin Yavari et al. [79] examined four SLM-produced Ti-6Al-4V ELI porous structures with porosities from 66% to 84%; the absolute S–N curves shifted widely with porosity, yet the S–N curves normalized to yield stress nearly coincided and followed a single power law (*R*^2^ = 0.94). Extrapolated to 10^6^ cycles, the normalized endurance limit of these structures falls to ~0.12—below that of bulk Ti-6Al-4V (~0.4), porous-coated Ti-6Al-4V (0.15–0.20), and EBM-produced porous Ti-6Al-4V (0.15–0.25) (comparison values compiled in Amin Yavari et al. [79]). Table 2 and Figure 3 collate and visualize these values.

**Table 2:** Normalized fatigue endurance limit (fatigue strength at 10^6^ cycles divided by the yield or ultimate strength reported by each source) for Ti-6Al-4V under different structural conditions. Normalization to strength allows for approximate comparison across studies with different absolute stress levels, but the entries are heterogeneous in porosity, R ratio, loading mode, surface condition, and normalization basis; the listed ranges are spans of the reported literature values, not statistical intervals. Values are as compiled and reported in the cited sources.

Structural Condition	Normalized Endurance Limit (~10^6^ Cycles)	Source
Bulk (wrought/solid) Ti-6Al-4V	~0.4	[79]
Porous-coated Ti-6Al-4V	0.15–0.20	[79]
EBM-produced porous Ti-6Al-4V	0.15–0.25	[79]
SLM-produced porous Ti-6Al-4V (66–84% porosity)	~0.12	[79]
Variable-diameter dense-core porous scaffold	~0.5 (at ~23 GPa effective modulus)	[1]

That the porosity-normalized S–N curves collapse onto a single power law indicates that, once normalized to strength, the fatigue behavior of these porous structures is governed by a common mechanism rather than by porosity per se [79]. The practical implication is visible in the table: conventional porous architectures sit well below bulk material, whereas a variable-diameter dense-core design has been reported—on the basis of a single value compiled in the review by Gao et al. [1], which would need independent confirmation—to recover a near-bulk normalized endurance limit while retaining a bone-compatible effective modulus. The low fatigue resistance stems chiefly from high surface roughness combined with the notch sensitivity of Ti-6Al-4V, manufacturing-derived thin or weak struts, and residual thermal stresses; this interplay of manufacturing defects, surface morphology, and fatigue behavior in additively manufactured Ti-6Al-4V strut-based lattices has been reviewed in detail [80]. Testing SLM-produced Ti-6Al-4V in three surface conditions (as-built, machined, and finely polished), Pagliari et al. [81] quantified how much surface roughness lowers the fatigue limit, sizing their specimens to represent the characteristic strut size of additively manufactured reticular structures. Masuo et al. [78] showed that, in additively manufactured Ti-6Al-4V, defects are mainly gas pores and lack of fusion; that removing near-surface pores by HIP raised the fatigue strength markedly, up to the ideal limit expected from the material’s hardness; and that surface roughness had a strong negative effect. Kumar and Ramamurty [82] likewise documented that the high-cycle fatigue behavior of SLM-produced Ti-6Al-4V is largely governed by process-induced porosity, surface irregularities, and microstructure. Fatigue failure typically follows a three-stage strain-accumulation pattern. The crack-initiation site matters too: Li and Affolter [71] reviewed that in additively manufactured Ti-6Al-4V the fatigue cracks originate mostly from internal defects (lack of fusion and gas pores), that about 85–90% of internal defects lie within 200 µm of the free surface, and that, because of their irregular, notch-like geometry, lack-of-fusion defects are more harmful than gas pores.

Still, Amin Yavari et al. [79] raise an important nuance: in bone-mimicking biomaterials, maximizing fatigue life is not always a justified design goal. As bone grows into the pores, the structure carries less load, and when the implant’s gradual loss of stiffness matches the rate of bone regeneration, this can even help translation. Fatigue is, therefore, best judged in its clinical context, not by the absolute rule of “the higher the better”. This reasoning applies chiefly to resorbable or regenerative scaffold concepts; however, a permanent load-bearing porous titanium implant, which does not degrade, must still retain an adequate component-specific fatigue safety margin throughout its intended service life, and bone ingrowth should not be taken to justify tolerating fatigue damage. It should be added that this is also how component qualification is framed. Endurance specifications of the kind set out in ISO 7206-4 and ASTM F3090 [75,76] are established on worst-case assumptions—the implant carrying the full load, without any supporting contribution from bone ingrowth, and in unfavorable component positioning—so that the qualified fatigue margin remains valid even where osseointegration is delayed or does not occur. Bone ingrowth, where it does occur, therefore, adds to that margin rather than being a precondition for it.

This trade-off can be managed through rational design. Beyond the dense-core route noted above, the node-free continuous surface of the TPMS-based structures noted in Section 4 improves the fatigue behavior by reducing the stress concentration. Lietaert et al. [77] showed that in AM Ti-6Al-4V lattices, fatigue failure concentrates where local tensile stress peaks at the strut nodes, and that in local-stress terms, single struts behave much like bulk Ti-6Al-4V—mechanically explaining the fatigue advantage of node-free continuous surfaces. Optimizing the pore size and distribution or adding dense-core regions, therefore, lets designers balance the mechanical strength, fatigue durability, and biomechanical compatibility.

Furthermore, “porous titanium fatigue” is not one phenomenon: an acetabular shell, a spinal cage, a metaphyseal cone, an augment, and a tumor prosthesis each work under a different loading regime (compression-dominant, bending, shear, torsion, and combinations of these). Fatigue testing should, therefore, be component-specific, reflecting the physiological loading profile of the part in question, and uniaxial-compression data should not be generalized to every component. In the load-bearing in vivo environment, moreover, corrosion-fatigue and fretting from micromotion (at screw–augment, cup–bone, and modular-junction interfaces) act alongside pure mechanical fatigue; yet these mechanisms remain an understudied gap for porous/additively manufactured titanium and should be examined in future work under physiological media (PBS/SBF).

For this review, fatigue carries special weight. It is one of the most clinically relevant yet inconsistently reported parameters in laboratory studies, yet it is the critical link between microstructural decisions and the lattice/strut-fracture failure mode seen in the clinic (see Section 7). Fatigue performance also depends on loading mode: Lietaert et al. [77] reported that tension–tension loading gives a shorter fatigue life than compression–compression loading, whereas most tests use uniaxial compression. Given the complex, tension-containing loads an implant experiences in vivo, compression-based data alone can make clinical durability look better than it is. When the fatigue cost of the porosity added for a low modulus goes unreported, the gap between laboratory success and clinical durability stays invisible.

A final point concerns what the in vitro fatigue data reviewed above can, and cannot, say about performance in a patient—an extrapolation whose limits deserve an explicit statement. Four gaps separate the coupon test from the clinical setting. The first is the loading mode: most published lattice data are uniaxial and compression-dominated, whereas physiological loading is multiaxial and includes the tensile and bending components under which lattices fail sooner, as the tension-compression asymmetry documented by Lietaert et al. [77] shows. The second is environment: fatigue testing is typically conducted in air at laboratory temperature, while the implant operates in a warm chloride-rich medium in which corrosion fatigue and fretting at interfaces act alongside pure mechanical fatigue—an interaction identified above as an understudied gap for porous titanium. The third is the load spectrum: laboratory S–N data are generated at constant amplitude to a defined run-out, whereas in vivo loading is a variable-amplitude history accumulated over years of gait, with occasional overloads, and the damage accumulated under such spectra is not reliably inferred from constant-amplitude runout behavior alone. The fourth is structural context: a coupon is tested as a free-standing structure, whereas a well-fixed implant shares load with ingrown bone and is qualified at component level under standardized worst-case assumptions (ISO 7206-4 [75] and ASTM F3090 [76])—assumptions that deliberately exclude the load-sharing benefit of ingrowth precisely because it cannot be guaranteed. The practical consequence is that in vitro lattice fatigue data are best read as comparative screening between architectures and process conditions, and component-level qualification as a regulatory floor; neither is a prediction of in-service human durability. The empirical link between the two can only be built from the clinical side, by reporting fatigue-relevant manufacturing and design parameters in clinical series (Table 5)—which, as Section 7.2 shows, is not yet being done.

## 6. Surface Engineering: Osseointegration and Antibacterial Strategies

The preceding sections focused on the bulk mechanical behavior of the porous structure—effective modulus, phase, and fatigue. Yet an implant’s clinical success rests not only on bulk properties but also on the surface that meets bone: the porous architecture supplies the right mechanical environment, but the rate and quality of bone ingrowth and the resistance to infection depend heavily on surface properties. Although titanium is regarded as bioinert, a recent study showed that healing macrophages in contact with the surface can be pushed toward a pro-inflammatory, tissue-destructive program that may contribute to late implant failure [83]—further underscoring the value of biologically optimizing the surface.

### 6.1. Surface-Modification Methods

Titanium implant surfaces can be treated by various physical and chemical methods to improve the bone–implant interaction. As synthesized by Li et al. [84], the main approaches include acid/alkali etching, anodization, micro-arc oxidation (MAO), chemical/physical vapor deposition (CVD/PVD) coatings, and friction stir processing (FSP). These methods regulate surface topography, chemistry, and roughness and so shape protein adsorption, cell attachment, and subsequent bone formation. In porous structures, a pore size of ~300–600 µm is commonly reported as favorable for bone ingrowth [66], a view confirmed in vivo: comparing SLM porous titanium implants with target pore sizes of 300, 600, and 900 µm in a rabbit model, Taniguchi et al. [66] found that the 600 µm structure gave the highest bone–implant fixation and the best ingrowth. Because higher porosity lowers mechanical strength, however, a porosity–strength trade-off must be respected [59]. The effects of design parameters, such as pore topology (periodic or stochastic), pore size, porosity, and strut diameter, on osseointegration have also been reviewed systematically [85]. These surface methods transfer well to additively manufactured titanium: Kozelskaya et al. [86] reported that micro-arc-oxidation calcium–phosphate coatings on AM titanium produce superior biocompatibility and osteogenic response (attachment, proliferation, and osteogenic differentiation) versus unmodified titanium, in vitro and in vivo. Xiu et al. [87] showed the method’s value in a porous load-bearing geometry directly: a microporous TiO_2_ and amorphous calcium–phosphate layer formed by single-step MAO on 3D-printed porous Ti-6Al-4V scaffolds sharply raised apatite-forming ability, cytocompatibility, and ALP activity and in a rabbit model turned the “distance osteogenesis” pattern—bone forming only at the periphery in untreated scaffolds—into deep, interlocked ingrowth. One of the most clinically important consequences of coating is fixation: Watanabe et al. [88] showed that a nano-hydroxyapatite (nHA) coating applied—without occluding the pores—to EBM-produced 3D porous Ti-6Al-4V implants increased bone ingrowth and early fixation (push-out strength) in a canine transcortical model, with potential to ease the radiolucent-line and weak-fixation concerns reported for EBM porous acetabular cups. In a recent comprehensive review, Yuan et al. [89] considered together the advanced surface-modification strategies aimed at the two main clinical problems that follow from titanium’s bioinert nature—insufficient osseointegration and susceptibility to bacterial infection—and stress that a single surface design often has to tackle both at once.

One constraint cuts across this entire group of methods and is specific to porous architectures. Plasma spraying—the most established route for depositing hydroxyapatite (HA) on orthopedic implants and the basis of the silver-doped coatings discussed in Section 6.2—is a line-of-sight process: the plume deposits where it can travel in a straight line from the torch, so it coats the outer surface of a component but cannot reach the internal strut surfaces of an open-cell lattice, and depositing enough material at the periphery risks occluding the entrance pores on which ingrowth depends. Plasma-sprayed HA is, thus, well matched to a solid substrate with a textured surface and poorly matched to the architecture reviewed here, whose functional surface is largely internal. The alternatives that do reach internal surfaces are those in which the coating forms from a liquid or gaseous phase that penetrates the pore network rather than being projected onto it. Micro-arc oxidation is the clearest example and is already documented in this review for exactly this geometry: Xiu et al. [87] used a single-step MAO treatment on 3D-printed porous Ti-6Al-4V to convert a peripheral “distance osteogenesis” pattern into deep, interlocked ingrowth, and Kozelskaya et al. [86] reported comparable results for MAO calcium–phosphate coatings on additively manufactured titanium. Other non-line-of-sight routes reported for porous titanium include alkali and heat treatment, which converts the surface itself into a bioactive titanate layer; biomimetic apatite deposition from simulated body fluid; electrochemical and electrophoretic deposition of calcium phosphates; sol-gel and hydrothermal deposition; and, where an ultrathin conformal layer is sufficient, atomic layer deposition [90,91]. Each carries its own limits—solution chemistry and current distribution are not uniform deep within a tortuous lattice, adhesion and layer thickness vary between methods, and none is exempt from the scale-up and regulatory constraints of Section 6.3—but all share the property that plasma spraying lacks: the coating forms on the internal surfaces where bone must actually grow. Watanabe et al. [88] make the corresponding design requirement explicit in the other direction, having applied a nano-HA coating specifically without occluding the pores and shown improved bone ingrowth and initial fixation. Coating method, coating thickness, and the uniformity of coverage between the peripheral and the deep regions of the lattice should, therefore, be reported as design parameters in their own right (Table 5).

### 6.2. Osseointegration and Antibacterial Function Through Bioactive Element Doping

Functionalizing the surface biochemically, not just topographically, is increasingly important. Surfaces doped with bioactive elements, such as zinc, magnesium, and copper, have shown promise both for stimulating bone formation and for suppressing bacterial colonization. As compiled by Zhang et al. [92], zinc-doped surfaces raised osteoblast proliferation by about 25% and cell adhesion by about 40% while inhibiting *Staphylococcus aureus* by about 24%, and magnesium-doped surfaces raised alkaline phosphatase (ALP) activity by about 38% and proliferation about 4.5 fold. Copper-doped surfaces showed an antibacterial efficacy of 99.45% against *S. aureus* and 98.65% against *Escherichia coli* [92]. One of the most established ways to deliver these elements to titanium is via a plasma-spray hydroxyapatite (HA) coating. Roy et al. [93] showed that plasma-sprayed silver-doped HA coatings on Ti kept their mechanical integrity, with an adhesion strength above 15 MPa, while resisting bacterial adhesion. Bioactive-element doping also aids osseointegration in vivo: Tao et al. [94] reported that HA-coated titanium implants containing zinc, magnesium, and strontium improved osseointegration in osteopenic rats. Beyond these in vitro findings, bioactive-element coatings are now being tested in load-bearing porous implants in vivo: Borgognoni et al. [95] assessed whether a strontium-containing Ti-Sr-O coating enhances early osseointegration of 3D-printed porous Ti-6Al-4V implants in a large-animal 2 mm gap model, tying this strontium approach to settings where early osseointegration is critical, such as oncologic reconstruction with compromised bone stock. More recently, Speijker et al. [96] showed that a silver multilayer coating cut bacteria by more than 99.2% against Gram-positive and Gram-negative strains—including clinical isolates from periprosthetic infection—on Ti-6Al-4V and Co-Cr surfaces in standardized tests (ISO 22196, JIS Z-2801, ASTM E-2180). It must be stressed, though, that most of these results come from in vitro or short-term animal models; while such surfaces reduce bacterial adhesion and biofilm formation in vitro, robust clinical evidence that they lower PJI rates in routine load-bearing arthroplasty is still limited.

The value of these strategies lies in their dual role: the same surface modification is aimed both at accelerating osseointegration to reduce aseptic loosening and at suppressing bacterial adhesion to lower periprosthetic infection. This second function ties directly to the infection failure mode discussed in the clinical section (see Section 7); antibacterial surfaces are especially valuable in load-bearing implants, where infection remains a leading cause of failure.

A distinction should be drawn before leaving this section. None of the strategies above is specific to porous architecture: doping, hydroxyapatite and silver coatings, and topographical modification can all be applied to conventional solid titanium surfaces, and most of the evidence cited for them was, in fact, generated on such surfaces. What is specific to the architecture reviewed here is not the countermeasure but the exposure—whether the much larger internal surface area of a printed lattice, as well as the crevices between its struts, themselves add to the baseline infection risk that every load-bearing implant carries. Three lines of evidence bear on this and point the same way without settling it. The surface area of a porous geometry is already established as a determinant of in vivo metal release, shown for porous titanium by Ducheyne et al. [97]. The as-built strut surface carries partially melted particles that raise bacterial adhesion and suppress osteogenic activity in vitro (Xie et al. [98]). And in the clinical setting, Abar et al. [99] attribute the difficulty of eradicating biofilm on custom porous implants to precisely this enlarged surface area and inter-strut crevice geometry. Set against this, no clinical study has isolated surface area as an independent risk factor: the series in which high infection rates are reported are also the series with revision status, prior periprosthetic joint infection, large defects, and compromised soft tissue, and these confounders are examined in Section 7.3. The honest position is, therefore, that the property porous architecture is designed to exploit (a large, accessible, interconnected internal surface) is biologically available to bacteria as well as to bone, that this is a mechanistically plausible and partly evidenced liability, and that it has not been quantified independently of host and surgical risk factors in any existing clinical series. Reporting the specific surface area of the porous region, alongside a uniform periprosthetic joint infection definition, would be the minimum needed to begin resolving it (Table 5).

### 6.3. Barriers to Application

Although most of these surface strategies perform strongly at the laboratory and cell-culture level, they meet real barriers on the way to the clinic. As Zhang et al. [92] stress, the main constraints are scalability (producing consistent coatings on large surfaces is hard), regulatory approval, and limited in vivo validation; much of the current evidence rests on cell-culture or short-term animal studies, and long-term clinical validation is still largely missing—the surface-engineering face of the review’s central laboratory–clinical gap, taken up further in Section 7 and Section 8.

## 7. Clinical Translation: Along the Axis of Failure Modes

**Figure 4:**
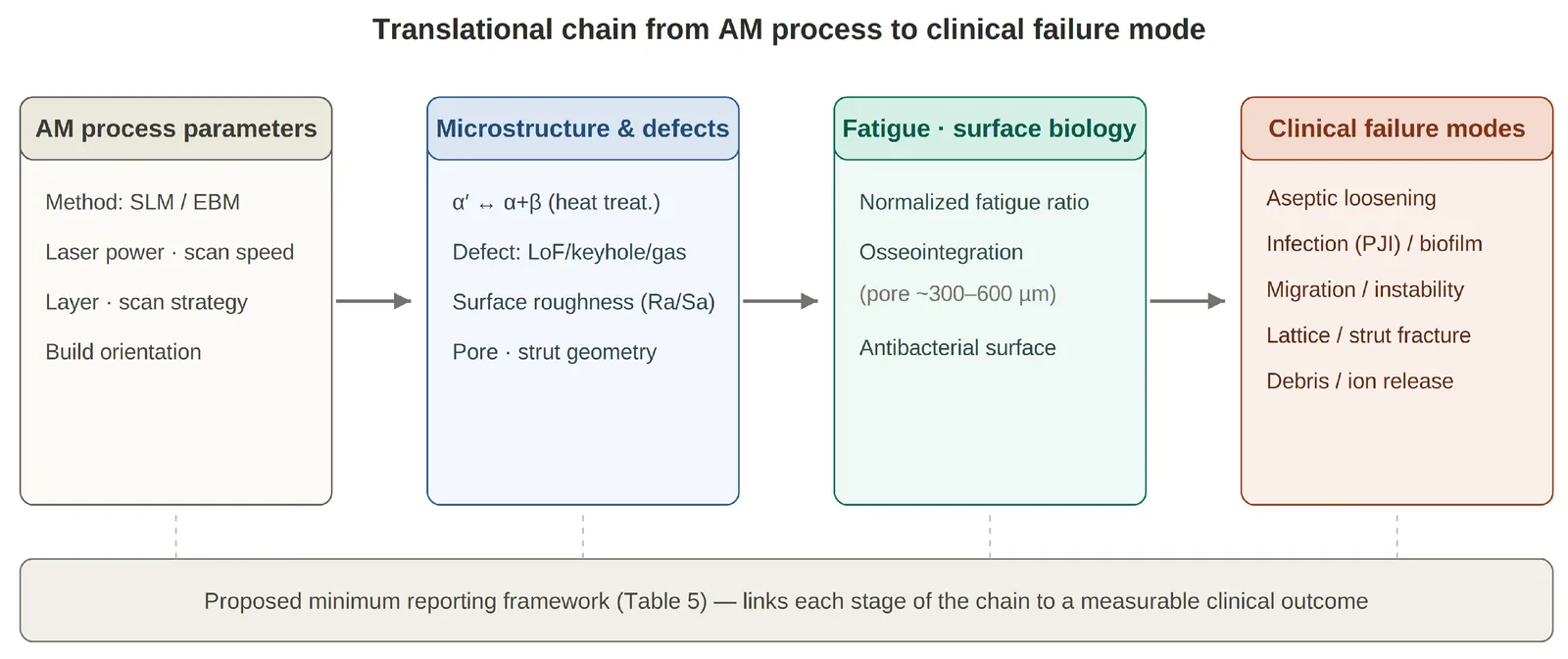
The translational chain extending from additive manufacturing (AM) process parameters to clinical failure modes. The material and manufacturing decisions at each stage (Section 4, Section 5 and Section 6) are linked to the clinical outcome (Section 7) through the next link; the proposed minimum reporting framework (Table 5) aims to ensure that each link of the chain is reported in a measurable and comparable manner.

The preceding sections dealt with the material, manufacturing, microstructure, and surface dimensions of porous titanium implants; this section synthesizes the ultimate test of all those decisions: clinical performance. Unlike most existing reviews, which organize clinical evidence by implant type or anatomical region, this one organizes it along the axis of clinical failure modes, tying each material or design decision directly to the clinical failure mode it is meant to address. Figure 4 shows the translational chain that captures this logic.

To make the logic of Figure 4 explicit rather than implicit, the causal chain behind each failure mode examined below can be stated in one line each. For lattice/strut fatigue fracture (Section 7.2), process parameters and powder feedstock determine the intra-strut defect population and as-built surface condition (Section 4.2); defects and surface notches act as local stress concentrators within struts loaded in bending and tension; cracks initiate preferentially at near-surface, irregular defects and propagate under cyclic load (Section 5.4); and the clinical manifestation is strut or component fracture. For infection (Section 7.3), the enlarged internal surface area and inter-strut crevices of the architecture, together with any residual partially melted particles (Section 4.2), increase the surface available for bacterial adhesion and complicate biofilm eradication, and the clinical manifestation is periprosthetic joint infection and infection-driven removal. For debris (Section 7.5), weakly bonded particles detach under load, generating third-body wear and an inflammatory stimulus, compounded by surface-area-dependent ion release. For aseptic loosening and osseointegration failure (Section 7.1 and Section 7.4), effective modulus, pore architecture, surface treatment, and primary stability jointly determine whether bone ingrowth or fibrous encapsulation follows, and micromotion above the ingrowth threshold tips the balance toward the latter. Each of these chains is mechanistically well supported at the laboratory end; what varies—and what this section examines mode by mode—is how far along each chain the clinical evidence actually reaches, and at which link the reporting gap of Table 5 interrupts the audit trail.

Before turning to the individual cohorts, one feature of this evidence base should be stated plainly: almost all of it sits at the lower end of the evidence hierarchy. The great majority of the studies are Level IV case series or single-arm retrospective cohorts; comparative (Level III) designs are rare, and randomized data are essentially absent. The survivorship and outcome figures below should be read in that light—as consistent, encouraging signals from predominantly low-level evidence rather than as high-certainty effect estimates. The clinical evidence centers on three core series read in full text: a multicenter 68-patient revision total hip arthroplasty (rTHA) series [2], an 18-patient Paprosky type III augment series [100], and a 3-patient custom-augment series combined with finite-element analysis [101]. Both of these augment series were performed in revision total hip arthroplasty. The Paprosky classification referred to throughout this section grades acetabular bone loss in revision total hip arthroplasty, with higher grades denoting progressively greater loss of supporting bone and, correspondingly, greater difficulty in achieving stable fixation. Two further acetabular cohorts read in full text support these: a 40-patient series using a fully porous shell with a cemented polyethylene liner [20] and a 154-patient series using the OsseoTi highly porous cup [102]. A sixth series, a 32-patient (32-hip) primary total hip arthroplasty cohort with minimum 7-year follow-up [103], is also read in full text. Table 3 summarizes these cohorts and the meta-analyses that pool them at the highest evidence level, along with selected series from non-acetabular applications. Because their biomechanical environments and primary endpoints differ, the table separates them by anatomical application block (A: hip/acetabular, B: spine, C: revision knee, D: tumor/segmental, and E: foot-ankle) and by evidence level. The narrative synthesis in this section is organized by failure mode, whereas Table 3 retains anatomical blocks to preserve clinical context; the two views are complementary.

**Table 3:** Summary of clinical evidence on porous titanium load-bearing implants, organized by anatomical application block and evidence level. THA: total hip arthroplasty; rTHA: revision THA; rTKA: revision total knee arthroplasty; ACDF: anterior cervical discectomy and fusion; PJI: periprosthetic joint infection; KM: Kaplan–Meier; HHS: Harris Hip Score; ODI: Oswestry Disability Index; AOFAS: American Orthopaedic Foot & Ankle Society score; PROM: patient-reported outcome; LLD: leg-length discrepancy; FEA: finite-element analysis; EBM: electron beam melting; RCS: re-entrant chiral structure; XLPE: highly cross-linked polyethylene. Evidence levels approximately: meta-analysis/pooled > comparative cohort (III) > case series/retrospective cohort (IV). Survivorship values are not directly comparable across rows: endpoint definitions (all-cause vs. aseptic-only), follow-up durations (1–8.25 years), and denominators differ substantially between series; they are presented to convey the direction and consistency of outcomes, not for head-to-head comparison. Bold rows are anatomical application-block headings; within a cell, an em dash separates the fields named in the corresponding column heading.

Study (Design)	Implant— Manufacturing—Material (*n*)	Indication— Follow-Up	Evidence Level	Survivorship/ Principal Failure Mode	Functional Outcome
**A. Hip/acetabular reconstruction—strongest clinical evidence**					
Berlinberg et al. (2022) [2]—retrospective cohort	Fully porous 3D-printed Ti acetabular shell (*n* = 68)	rTHA, Paprosky ≥2A 94%—2 years	IV	All-cause 81%, aseptic shell 90%; PJI ~10–12%, instability 3%, no migration	HHS 49.9 → 80.2
Fu et al. (2022) [100]—case series	3D-printed porous Ti augment (*n* = 18)	rTHA, Paprosky III—33.3 months	IV	No radiographic loosening/dislocation/PJI	HHS 40.3 → 88.4; LLD 31.7 → 7.5 mm
Shi et al. (2025) [101]—case series + FEA	Custom porous Ti augment (*n* = 3)	rTHA, Paprosky III—short term	IV	No loosening/migration; peak stresses markedly reduced in FEA	HHS ~33–46 → ~88–89
Shichman et al. (2022) [20]—retrospective cohort (KM)	Fully porous Ti cup + XLPE liner (*n* = 40)	6 complex primary + 34 revision—KM	IV	KM 1-year 95%, 4-year 92%; aseptic migration 2.5%, PJI 5%; osseointeg. 97.5%	HHS 53.9 → 83.5
Bornes et al. (2026) [102]—retrospective cohort	OsseoTi highly porous cup, ~475 µm (*n* = 154)	71 primary + 83 revision—4 years	IV	Overall 88.2%; primary 100%, revision 79%; 2 aseptic loosenings (revision)	Radiographic possible loosening: primary 2.2%/revision 3.8%
Huang et al. (2021) [103]—multicenter cohort	EBM porous-coated trabecular Ti cup (*n* = 32)	Primary THA—≥7 years	IV	KM cup survivorship 96.3% at 8.25 years; all cups osseointegrated	—
Almeida et al. (2025) [17]—systematic review + meta-analysis	3D-printed/trabecular Ti acetabular cup; 11 studies (597 joints/585 patients)	rTHA—3.8 years	Pooled (meta)	All-cause 95.52%; aseptic loosening 0.8%, PJI 2.8%, instability 2.3%, migration 0.3%	HHS 86.7
**B. Spinal fusion cages—established use; different endpoint (fusion/subsidence)**					
Calek et al. (2026) [104]—retrospective cohort	3D-printed porous Ti interbody cage (*n* = 100; 137 levels)	Anterior/lateral lumbar fusion—1 year	IV	Fusion 97.1%; no subsidence/migration revision	ODI 39 → 10
Singh et al. (2024) [16]—comparative cohort	3D-printed porous Ti cage vs. allograft	ACDF—mid term	III (comparative)	Significantly less subsidence with titanium; similar fusion and PROM	Similar to allograft
**C. Revision knee metaphyseal cones—case-series and pooled evidence**					
Shichman et al. (2023) [21]—multicenter case series	Porous Ti metaphyseal cone	rTKA, metaphyseal defect—mid term	IV	Good radiographic/functional outcome	—
Mancino et al. (2022) [18]—systematic review	3D-printed porous Ti metaphyseal cone; 7 studies (687 cones/557 knees)	rTKA—~24 months	Pooled (meta)	All-cause 95.3%; free of aseptic loosening 99.7%	—
**D. Tumor/segmental reconstruction—patient-specific, low *n*, early term**					
Tan et al. (2025) [105]—case series	Patient-specific 3D-printed hemipelvic prosthesis, RCS porous (*n* = 15)	Pelvic tumor resection—mid term	IV	86.7% disease-free survival (oncologic outcome, not implant survivorship)	Functional improvement
Yon et al. (2025) [106]—case series	EBM patient-specific Ti-6Al-4V prosthesis (*n* = 3)	Foot/ankle tumor resection—short term	IV	Early bone integration	Marked AOFAS improvement
**E. Foot/ankle critical-size defects—infection-dominant, custom implant**					
Abar et al. (2022) [99]—case series	Custom 3D-printed porous Ti-6Al-4V implant (*n* = 39)	Critical-size foot/ankle bone defect—mid term	IV	Overall infection 15%; ~60% of removals due to infection	—

### 7.1. Aseptic Loosening, Stress Shielding, and Osseointegration Failure

This failure mode is central to the rationale for porous implant architecture, and the clinical evidence in this mode is favorable. In the 68-patient series of Berlinberg et al. [2]—in which 94% of patients had a Paprosky 2A or greater defect—2-year revision-free survivorship was reported as 81% for all causes, 88% for acetabular revision, and 90% for aseptic shell failure; all shells that were not removed showed signs of osseointegration, none showed migration, and the Harris Hip Score (HHS) rose from a mean of 49.94 to 80.15. Fu et al. [100], reporting on 18 patients, saw no radiographic loosening or radiolucency at a mean follow-up of 33.3 months; the HHS rose from 40.3 to 88.4, the leg-length discrepancy (LLD) decreased from 31.7 mm to 7.5 mm, and the vertical position of the hip center of rotation (HCOR) decreased from 50.7 mm to 22.3 mm. In the three patients of Shi et al. [101], the HHS improved from 32.8 to 88.1, from 43.2 to 87.6, and from 46.2 to 89.3, with marked correction of the LLD.

This clinical picture is also supported mechanically by finite-element findings: Shi et al. [101] showed that the custom porous augment reduced the peak stresses on the cup, screws, and pelvis compared with a commercial control under all load conditions; for example, under a six-times-body-weight (running) load, the maximum principal stress in the acetabular cup ranged from 20.35 to 89.58 MPa across the three cases, versus 175.1 MPa in the commercial control.

The two additional acetabular cohorts examined in full text reinforce this pattern. Shichman et al. [20] treated 40 patients (6 complex primary, 34 revision) with a fully porous Ti-6Al-4V shell with a cemented highly cross-linked polyethylene liner, the HHS rose from a mean of 53.87 to 83.53, and 39/40 (97.5%) of the components osseointegrated without migration; Kaplan–Meier all-cause revision-free survivorship was 95% at 1 year and 92% at 4 years, and survivorship free of aseptic shell/liner/fracture was 97.1% at 2 years. In the 154-patient series of Bornes et al. [102] (71 primary, 83 revision) using the OsseoTi highly porous cup (Ti-6Al-4V, ~475 µm pores), acetabular revision-free survivorship at 4 years was 88.2% overall—100% in primary arthroplasty and 79% in revision—with only two aseptic loosening failures (both in revision THA). As reported within Shichman et al. [20], high survivorship has also been reported in similar cohorts (e.g., 98.4% aseptic survivorship at a mean of 87.6 months in 62 rTHA cases; 99.1% at 2 years in a complex primary cohort). Berlinberg et al. [2] also report, in their discussion and based on data compiled from other series, that porous titanium and tantalum shells show survivorship in the range of 98–100% up to 7 years, with migration or loosening in almost no patients. These particular figures are secondary (compiled within [2] rather than measured in that cohort) and, like several of the strongest acetabular series, originate partly from overlapping author groups (see Section 9); they should, therefore, be read as indicative rather than as independent pooled estimates. This long-term pattern is also directly supported by a primary cohort examined in full text: Across five centers, Huang et al. [103] followed 32 primary total hip arthroplasty patients with EBM-produced porous-coated trabecular titanium cups for at least 7 years. Kaplan–Meier cup survivorship reached 96.3% (CI 76.5–99.5%) at 8.25 years, and every cup that was not removed achieved osseointegration. Specific to primary arthroplasty, this finding partly answers the criticism that long-term evidence for porous architecture is scarce.

A recent systematic review and meta-analysis pooling these individual cohorts at the highest evidence level confirms the same direction: Almeida et al. [17], pooling 11 studies covering 597 joints in 585 patients, found an all-cause survivorship of 95.52% (CI 92.37–97.96%) at a mean follow-up of 3.8 years and a mean postoperative Harris Hip Score of 86.7 for porous 3D-printed Ti acetabular cups; among the reasons for reoperation, aseptic loosening was only 0.8% (infection 2.8%, instability 2.3%, and shell migration 0.3%).

This pattern is not limited to the hip; similar results have been reported in revision knee arthroplasty. Mancino et al. [18] pooled seven studies and 687 metaphyseal cones used in 557 revision knees; the all-cause revision-free survivorship of 3D-printed porous titanium cones was 95.3% at a mean of 24 months and survivorship free of aseptic loosening was 99.7%; it was concluded that these cones provide high fixation and offer lower intraoperative complications and higher survivorship free of aseptic loosening than tantalum cones. Similarly, patient-specific 3D-printed porous titanium cones have been shown to be usable in bone-defect management by preserving the remaining bone stock in complex primary and revision knees [107].

Taken together, these data suggest that the porous Ti-6Al-4V architecture is associated with favorable performance in the outcomes that clinical cohorts directly measure—high osseointegration and low aseptic-loosening rates—rather than with a directly measured reduction in stress shielding (see below). Even so, especially in revision cases, aseptic loosening is not entirely eliminated, and survivorship varies by cohort and indication (e.g., 79% at 4 years in the revision subgroup of Bornes et al. [102]).

This clinical pattern also aligns with controlled preclinical evidence: Feldman et al. [108] showed by histomorphometry, histopathology, and micro-CT that additively manufactured Ti-6Al-4V lattice implants achieved extensive and comprehensive osseointegration by 12 weeks in a bicortical load-bearing ovine femur model, with bone maturation still ongoing at study end. In a longer preclinical study, Palmquist et al. [109] evaluated free-form porous and solid Ti-6Al-4V implants produced by electron beam melting (EBM) in a sheep model over 26 weeks and reported that both structures osseointegrated. That engineered architecture can directly improve osseointegration has likewise been demonstrated in vivo: Li et al. [110] evaluated gradient porous Ti-6Al-4V scaffolds designed with triply periodic minimal surface (TPMS) models in a minipig model, showing that these structures reduced stress shielding and increased bone–implant bonding strength by stimulating early osteogenesis and osseointegration. This finding supports the architecture-based approach by providing the biological correlate of the mechanical advantages of rational TPMS design (see Section 4.3). A qualification applies to this preclinical evidence as a whole. The implants used in these studies were purpose-made test constructs placed in defect, transcortical or bicortical models—not clinical prostheses under physiological joint loading. They, therefore, establish that a given architecture or surface can osseointegrate under controlled conditions, but they do not reproduce the articulation, the loading history, or the compromised bone quality of the revision setting in which these devices are actually used. This is one reason the preclinical and clinical columns are kept separate in the synthesis matrix of Table 4.

Nevertheless, it is important to make an evidence-level distinction in this failure mode. The two outcomes that clinical series directly and strongly support are high osseointegration rates (radiographic evidence) and low aseptic-loosening rates (cohort survivorship evidence). By contrast, the claim that “stress shielding is reduced” has not been directly measured in most clinical series; this inference rests on finite-element analyses (e.g., Arabnejad et al. [59]) and preclinical in vivo models (e.g., Li et al. [110]). Clinical data directly demonstrating preservation of periprosthetic bone mineral density by serial DEXA/BMD or quantitative computed tomography are relatively limited. Therefore, the conclusion that porous architecture reduces stress shielding clinically should be regarded as a plausible but largely mechanistic and preclinically based inference; while the clinical survivorship and osseointegration data are consistent with this inference, they are not its direct proof.

The distinction the preceding paragraph draws can be taken one step further, because the clinical evidence on stress shielding itself—independent of the porous implants reviewed here—follows a consistent three-part pattern. First, periprosthetic bone loss after cementless hip arthroplasty is real, measurable, and well characterized by longitudinal dual-energy X-ray absorptiometry (DEXA). Bone mineral density (BMD) loss concentrates in the proximal femur and, above all, in the calcar (Gruen zone 7), develops predominantly within the first months after implantation, and is modulated by stem stiffness; in a prospective two-year DEXA series of female patients, Alm et al. reported that total periprosthetic BMD fell by ~3.7–3.8% within the first six months, while zone 7 showed a persistent 23% decrease at 24 months [111], and a systematic analysis of published DEXA data by Knutsen et al. located the greatest resorption in zone 7 across stem designs, with stiffer cobalt–chromium stems producing roughly twice the proximal bone loss of titanium-alloy stems [112]. Second—and this is the step that matters for the design rationale—measured periprosthetic bone loss is a surrogate outcome whose link to patient-level failure has not been clearly demonstrated in the studies reviewed here. In 208 extensively porous-coated total hip arthroplasties followed for a mean of 13.9 years, Engh et al. found that the hips with pronounced radiographic stress shielding developed no femoral loosening, implant fracture, or loss of porous coating, and their reported 15-year survivorship was, in fact, higher than that of hips without stress shielding (93% vs. 77%); the authors concluded that the stress shielding “did not produce adverse consequences” in these arthroplasties [113]—an observation consistent with pronounced stress shielding arising precisely in stems that are rigidly osseointegrated. Randomized evidence points the same way from the opposite direction: meta-analyses of trials comparing short or proximally loading stems with conventional stems report better preservation of zone 1 and zone 7 BMD with the less stiff designs—significant overall in one analysis [114] and in design subgroups in another [115]—yet either found no difference in the revision rate [114] or could not assess revision at all because the follow-up was too short [115]. Third, none of this renders stress shielding clinically irrelevant, because its cost appears in outcomes other than aseptic loosening: proximal femoral bone resorption was identified as an independent risk factor for postoperative periprosthetic femoral fracture in a large retrospective arthroplasty cohort [4], and periprosthetic bone loss erodes the bone stock on which any later revision depends, making reconstruction technically more demanding [5]. For this review, the implication is uncomfortable but clarifying. The stress-shielding rationale for porous architecture rests on a chain—lower stiffness, therefore, preserved periprosthetic bone, therefore, fewer failures—whose first link is well supported, whose second link is documented for conventional stem designs, and whose third link has not, to date, been directly demonstrated for any design. For the additively manufactured porous implants reviewed here, even the second link is missing: to our knowledge, none of the clinical series summarized in Table 3 has reported serial DEXA or other quantitative bone-density outcomes. The biomechanical concept of stress shielding is, thus, well established, while its translation into a demonstrated clinical benefit of low-stiffness design has yet to be established—the same laboratory-to-clinic asymmetry this review traces for the other design parameters (Table 4).

### 7.2. Lattice/Strut Fatigue Fracture

This mode is the clinical counterpart of the fatigue trade-off from Section 5. Strut fracture is a theoretical risk in porous structures, yet it is rarely reported in clinical series. In their finite-element analysis, Shi et al. [101] found the peak stresses in the custom augment stayed well below the engineering flexural strength of the titanium alloy (889–921 MPa), pointing to a low risk of strut fracture in the short to mid term. The core problem, though, is that fatigue performance is almost never reported directly in clinical series, so the true fracture risk under long-term cyclic loading remains largely unknown. This reporting gap is one of the clearest examples of the disconnect between laboratory fatigue data (Section 5.4) and clinical durability. The distinction drawn in Section 5.4 sharpens what is actually missing. Where strut fracture does occur, the mechanism implicated by the laboratory evidence is crack initiation at an intra-strut defect or a surface notch rather than an intrinsic consequence of the designed porosity—which means that the determining variables are defect quantity, surface condition, and post-processing status, none of which the clinical series report. The low observed rate of strut fracture is, therefore, reassuring about current devices as used, but it cannot be read as evidence that the architecture is inherently fracture-resistant, because the variables that would govern that conclusion are not documented in any of the series reviewed here.

### 7.3. Infection and Biofilm

Periprosthetic joint infection (PJI) is a leading cause of failure for load-bearing implants of every kind—cemented and cementless, solid-surfaced and porous—and is not a complication specific to porous titanium. What may be specific, and what this section examines, is whether the substantially larger internal surface area of a printed porous architecture adds to that baseline risk. In the series of Berlinberg et al. [2], three shells (4%) were removed early (at months 1, 3, and 6) for PJI, and the total PJI rate was about 10–12%. This is above the 3–5% previously reported in porous implant series, but the authors note that 22% of the cohort had a prior history of PJI, and that this complicated profile raised the rate. By contrast, no PJI occurred in the smaller, primary-defect-dominated series of Fu et al. [100]. Shichman et al. [20] re-revised two patients (5%) with debridement, antibiotics, and implant retention (DAIR) for acute PJI; the fact that 32% of the revision cases here presented with PJI as the indication supports the view that a prior infection history perpetuates the risk. There is also direct clinical evidence that porous architecture may specifically complicate infection management. Abar et al. [99] found an overall infection rate of 15% in a 39-patient series in which custom 3D-printed porous Ti-6Al-4V implants were used for critical-size foot and ankle defects, with infection driving implant removal in most cases (~60%). The authors argue that biofilm eradication may be harder because of the much larger surface area of 3D-printed implants and the crevices between their struts—so the very property porous structures seek for osseointegration can become a liability against infection. Causality, however, must be read cautiously: PJI is driven mostly by patient profile, prior infection history, revision status, defect size, surgical duration, and soft-tissue condition, and the high-rate series above are laden with such confounders—prior or concurrent PJI in 22% of Berlinberg’s cohort, for instance, and in 32% of Shichman’s revision cases. One, therefore, cannot conclude that porous architecture independently raises infection risk; the more cautious reading is that the porous surface may offer more area for bacterial retention and complicate biofilm eradication, while current series remain confounded by revision status, prior PJI, defect severity, and host factors. This mode connects directly to the antibacterial surface strategies of Section 6: although zinc- and copper-doped surfaces are strongly antibacterial in vitro, they have not yet been integrated into routine load-bearing arthroplasty. Silver-based coatings, by contrast, have entered limited clinical use in selected high-risk cases and tumor megaprostheses (e.g., silver-hydroxyapatite-coated cementless hip prostheses and silver-coated megaprostheses) and are beginning to gather early cohort and case support. In one such series, Kawano et al. [116] reported 5-year results in 50 patients receiving a silver-oxide-containing hydroxyapatite (Ag-HA)-coated cementless total hip prosthesis at high infection risk, combining the antibacterial action of silver with the osteoconductivity of hydroxyapatite. Likewise, in oncologic megaprosthesis reconstruction, where periprosthetic infection risk is very high, systematic reviews and meta-analyses report that silver-coated prostheses can lower infection rates [117]. Even so, infection remains an area where laboratory success in surface engineering has yet to find its routine clinical correlate.

### 7.4. Fixation Loss, Migration, and Instability

Early stability and long-term fixation are critical to the success of porous implants. In the series by Berlinberg et al. [2], two patients (3%) were revised for instability/dislocation, but none of the retained shells migrated; the authors credit the high-friction surface and angled locking screws for the early stability. No dislocation occurred in the series by Fu et al. [100]. Shichman et al. [20] saw aseptic loosening with proximal shell migration in just one case (2.5%), and 97.5% of components osseointegrated without migration; Bornes et al. [102] reported possible radiographic loosening in 2.2% of primary and 3.8% of revision THA. Overall, the early mechanical grip of the porous structure and the subsequent bone ingrowth address this mode reasonably well, and instability cases relate mostly to joint biomechanics and surgical positioning rather than to the implant–bone interface.

### 7.5. Debris and Partially Melted Powder Particles

This manufacturing-derived mode is a risk specific to additive manufacturing. As Li et al. [84] note, partially melted powder particles weakly bonded to the strut surface can detach over time, causing third-body wear and debris. Xie et al. [98] showed in vitro that partially melted particles on 3D-printed Ti-6Al-4V implants increased bacterial adhesion and suppressed osteogenic activity—evidence that these particles carry a risk not only of mechanical wear but also for infection and osseointegration. Beyond particulate debris, the porous geometry itself has a corrosion/ion-release dimension: measuring the trace-metal levels released into surrounding tissue 6 and 12 months after implanting solid and porous Ti and Ti-alloy specimens into canine trabecular bone, Ducheyne et al. [97] showed early on that porous geometry, through its large surface area, is an important determinant of in vivo metal-ion release. Once again the large surface area porous architecture seeks for osseointegration can carry a cost, paralleling the biofilm concern in Section 7.3. The mode ties directly to the manufacturing defects and surface post-processing of Section 4.2 (loose-powder removal, HIP); though rarely reported directly in clinical series, it underscores the importance of manufacturing quality control.

### 7.6. Evidence Maturity by Anatomical Application

The failure modes above are best documented in hip-acetabular reconstruction, but the maturity of clinical evidence for porous titanium varies markedly by application region.

Acetabular reconstruction is the most mature area, with cohort-level survivorship and osseointegration data (Section 7.1). In spinal surgery, porous titanium is by now an established practice: a 100-patient (137-level) lumbar fusion series using 3D-printed porous titanium interbody cages reported a 97.1% fusion rate at 1 year with no revisions for subsidence or migration [104], and at the cervical level, 3D-printed porous cages subsided significantly less than allograft while matching it on fusion rates and patient-reported outcomes [16]. Even so, spinal evidence rests mostly on radiographic fusion endpoints; comparative long-term survivorship cohorts of the acetabular kind remain few. In knee revision, porous titanium metaphyseal cones have performed well at the case-series level [21]. In tumor and segmental reconstruction, the evidence is mostly small patient-specific series plus biomechanical work. Yon et al. [106] reported marked functional improvement and early bone integration in a three-patient foot-and-ankle series, patient-specific 3D-printed pelvic implants have been reported after tumor resection [118], and Tan et al. [105] used custom hemipelvic prostheses with a re-entrant chiral porous structure in 15 patients—a design aimed squarely at the trade-off, central to this review, between raising porosity for ingrowth and preserving mechanical stability (see also [3,9]).

McCloskey et al. [3] point out that patient-specific porous implants still sit in the shadow of off-the-shelf implants, owing to the latter’s lower cost, documented long-term durability, reimbursement coverage, and stronger clinical evidence base. In non-acetabular applications, then, the gap between laboratory/biomechanical promise and clinical validation is wider still—another observation that reinforces the review’s central thesis.

Given that this evidence base is predominantly Level IV, it is fair to ask why any inference of favorable clinical survivorship is drawn from it at all, and the answer should be stated together with its boundaries. The inference rests on four mutually reinforcing observations rather than on any single series: the direction of the findings is consistent across cohorts from different institutions, countries, and manufacturers (Table 3); the pooled estimate of the systematic review and meta-analysis [17] points the same way; a registry-level comparison against established uncemented cups found no significant difference in the revision rate [119]; and the osseointegration signal has an independent preclinical correlate (Section 7.1). Convergence of this kind is the strongest conclusion low-level evidence can support—but it supports a deliberately narrow one. The claim justified by these data is that porous Ti-6Al-4V implants show favorable short- to mid-term survivorship in the indications actually studied, dominated by revision acetabular reconstruction in experienced centers. It is not a claim of superiority over established alternatives (Section 7.7), of long-term durability beyond the follow-up actually accrued, or of generalizability to indications, surgeons, or health systems outside those studied. The principal threats to even the narrow claim—endpoint heterogeneity, short follow-up, overlapping authorship (Section 2.2), and the susceptibility of small single-arm series to selective reporting—are the reasons this review grades and appraises the evidence formally (Tables S3 and S4) rather than narrating it alone.

### 7.7. Fully Porous Constructs and the Comparison with Established Cementless Technologies

Two related questions cut across the evidence reviewed above: how much of it concerns implants that are actually porous through their load-bearing volume and how the performance of additively manufactured porous implants compares with the cementless technologies that preceded them.

On the first question, a structural distinction should be made explicit. In most primary acetabular components, the porous region is an ingrowth interface carried on a solid hemispherical shell, so that the stiffness of the component is set by the solid substrate and the porous layer functions as a fixation surface—the coupon-versus-component distinction already drawn for Figure 2. At the other end of the spectrum stand constructs in which the porous architecture is the load-bearing volume itself: metaphyseal cones and sleeves in revision knee arthroplasty [18,107], porous augments in acetabular revision [100,101], interbody fusion cages [16,104], and the fully porous revision shells reviewed in Section 7.1 [2,20]. This class is not new: porous tantalum metaphyseal cones for severe tibial bone loss were reported by Meneghini et al., in 2008, with all fifteen cones osseointegrated and no loosening or migration at short-term follow-up [120], building on the tantalum lineage introduced in Section 3.3. For these predominantly porous constructs, the architecture is not an interface property but the implant itself, and the fatigue, defect, and powder-cleaning considerations of Section 4 and Section 5 apply to the full load path rather than to a surface layer.

On the second question, the evidentiary bar set by established cementless technologies is high. Conventional, non-additively manufactured porous-coated components have decades of follow-up: a 19-to-25-year series of the Harris–Galante cup reported 86.8% survivorship at a mean of 22.5 years [121], and plasma-sprayed hydroxyapatite-coated stems perform at least as well, the Corail stem showing 96.8% stem survivorship at 20 years [122]. Porous tantalum, the most established highly porous fixation material [48], adds a cautionary registry lesson: in a collaborative analysis of the Swedish and Australian arthroplasty registries comprising 93,709 hips, trabecular-metal cups showed a higher adjusted risk of revision than other uncemented cups in primary arthroplasty (8-year survivorship of 94.4% versus 96.2%; hazard ratio of 1.5), with no protective effect against revision for infection [123]. A more bone-like porous fixation surface did not, in other words, outperform conventional cementless fixation in the primary setting where conventional fixation already works reliably.

Against this background, the direct comparative evidence for 3D-printed porous titanium remains limited and, so far, points to comparability rather than superiority. In the New Zealand joint registry, 2192 3D-printed primary acetabular cups showed a revision rate statistically comparable to that of 39,080 established uncemented cups (0.77 versus 0.55 per 100 component-years; hazard ratio of 1.29, *p* = 0.058) [119]. In the spine, a retrospective comparison of 3D-printed porous titanium with conventional titanium interbody cages reported lower subsidence (5.88% vs. 9.88%) and higher anterior fusion rates at 24 months [124]—one of the few comparative signals in favor of printed architectures, though radiographic rather than survivorship based. In our search, we identified no randomized trial directly comparing 3D-printed porous implants with conventional cementless or hydroxyapatite-coated implants in any load-bearing application. Taken together, the available comparative evidence does not yet show a measurable clinical advantage for printed porous titanium over established cementless technologies in primary arthroplasty, where the best-performing established designs reach ~97% survivorship at 20 years [122]; its more defensible advantage appears to lie where established off-the-shelf implants offer no good option—severe acetabular bone loss, Paprosky type III defects, custom augments, and tumor reconstruction—and there the advantage is geometric (patient-specific fit and defect filling) rather than a demonstrated modulus benefit. This reading is consistent with the observation of McCloskey et al. [3] in Section 7.6, and it is itself a statement of the translational gap this review describes: the architectural route has largely solved a manufacturing problem, while the comparative clinical question has, so far, yielded no evidence of superiority.

Polyetheretherketone (PEEK) completes this comparative picture, because in the interbody-cage segment it—rather than any metal—is porous titanium’s principal competitor. PEEK offers an elastic modulus of roughly 3–4 GPa, close to the trabecular bone, together with radiolucency that simplifies fusion assessment [125,126]; its weakness is the mirror image of titanium’s strength: the polymer is biologically inert, and without surface modification, it tends toward fibrous encapsulation rather than direct bony integration [126]. The clinical comparison with 3D-printed porous titanium cages is, accordingly, one of the few places in this review where head-to-head evidence exists. A meta-analysis of lateral lumbar interbody fusion found significantly lower cage subsidence with 3D-printed porous titanium than with PEEK (odds ratio of 0.25, 95% CI 0.14–0.44) [127], while a meta-analysis in anterior cervical fusion found no significant difference in fusion rate (odds ratio of 1.74, 95% CI 0.71–4.27) alongside fewer complications and better maintenance of segmental geometry with the printed titanium cages [128]. Read against the comparability findings above, this comparison sharpens rather than changes the conclusion: where porous titanium shows a measurable comparative advantage, it is against a bioinert polymer on interface-dependent endpoints (subsidence, geometry), not against established cementless metal fixation on survivorship.

### 7.8. Comparison of Laboratory Claim and Clinical Evidence

The failure modes above can be pulled together into a single framework. Table 4 makes the gap visible by placing the laboratory claim and the clinical evidence side by side for the principal design parameters; the minimum reporting parameters flagged in each row are then expanded into a full checklist (Table 5) in Section 8.3. The two tables do different jobs: Table 4 is a diagnostic matrix that identifies the laboratory–clinical gap for each design parameter, whereas Table 5 is a prescriptive recipe for what should be reported to close it.

**Table 4:** Comparison of laboratory rationale and clinical evidence on a design-parameter basis for porous titanium load-bearing implants; each row is linked to the related failure mode, evidence level, translational gap, and minimum parameter to be reported. LoF: lack of fusion; Ra/Sa/Sz: surface-roughness metrics. The symbol ↓ denotes a reduction; in the column heading, an em dash separates the two fields reported in that column.

Design Parameter	Laboratory Rationale—Key Experimental Evidence	Clinical Correlate	Related Failure Mode	Evidence Level	Translational Gap	Minimum Parameter to Report
Elastic modulus matching	β-Ti ~40 GPa in bulk, single-digit GPa when porous; architecture matches modulus to bone (Arabnejad et al. [59]: stress shielding ↓~75%)	Low effective modulus provided by porous Ti-6Al-4V architecture, not β-Ti; high osseointegration/low loosening in cohorts	Aseptic loosening/stress shielding	FEA + cohort (modulus indirect)	Periprosthetic BMD loss well documented for cementless stems (DEXA) but not linked to patient-level revision; no serial DEXA in porous-implant cohorts; β-Ti not translated to common clinical implants	Measured effective elastic modulus; relative density
Pore size/porosity	~300–600 µm commonly favored for biology, ~500–800 µm for modulus (Taniguchi et al. [66]: 600 µm best fixation)	Osseointegration reliable (~90–100%), migration rare	Osseointegration/fixation loss	Preclinical in vivo + cohort	Designed-vs-measured pore discrepancy; quoted ranges are application-specific, with no single optimum supported by comparative clinical evidence (Section 4.3); no inter-manufacturer reporting standard	Designed and micro-CT-measured pore size, porosity (%), strut diameter, interconnectivity
Unit-cell type (TPMS/strut)	TPMS node-free continuous surface → more uniform stress, better fatigue/permeability (Bobbert et al. [65]; Lietaert et al. [77])	Cell type rarely reported in clinical series	Lattice fatigue/osseointegration	Biomechanical + preclinical	Architecture type not stated in clinical series; no direct clinical comparison	Unit-cell type/topology and design source
AM defect control/surface roughness	Defect size/location and roughness determine fatigue; HIP improves (Tammas-Williams et al. [52]; Masuo et al. [78])	Defect quantity/HIP status rarely reported in clinical series	Lattice/strut fracture/debris	Experimental + preclinical	Defect quantity and post-processing not reported in clinical series	Defect quantity (LoF, keyhole, gas pore); Ra/Sa/Sz; HIP and post-processing status
Fatigue behavior	Porous normalized fatigue ratio ~0.12–0.25 (Amin Yavari et al. [79]); dense-core ~0.5 (compiled in [1])	Strut fracture rarely reported clinically	Lattice/strut fracture	Biomechanical/preclinical; sporadic case	Fatigue almost never reported in clinical series; component-specific testing lacking	Component-specific fatigue protocol; R ratio, frequency, environment, run-out cycles, normalized fatigue ratio
Surface bioactivation	Zn/Mg/Cu, HA, MAO enhance osseointegration (Xiu et al. [87]; Watanabe et al. [88])—mostly in vitro/animal	Infection still 4–15%; bioactive surfaces not integrated into routine implants	Osseointegration/infection	In vitro/animal	Integration of bioactive surfaces into load-bearing clinical implants and in vivo validation lacking	Surface treatment; evidence level (in vitro/in vivo/clinical)
Antibacterial efficacy	In vitro > 98% (Cu); silver coating > 99% reduction (Zhang et al. [92]; Speijker et al. [96])	PJI remains a leading cause of failure; limited clinical (silver megaprosthesis)	Infection (PJI)	In vitro; limited clinical	In vitro adhesion ↓ ≠ clinical PJI ↓; evidence insufficient in routine arthroplasty	PJI definition; antibacterial agent, test standard, evidence level

### 7.9. Resolving the Disconnect

The pattern that Table 4 reveals speaks to the question posed in Section 1. In current clinical practice, the low effective modulus is achieved through the porous architecture of conventional Ti-6Al-4V rather than through the low-modulus β-Ti alloys developed in materials science. As McCloskey et al. [3] document, most additively manufactured orthopedic implants in clinical use are Ti-6Al-4V-based—thanks to its well-established biocompatibility, its processability on SLM/EBM platforms, its relatively low cost, and its mature fatigue and defined regulatory pathways—while low-modulus β-Ti alloys, which have not yet reached the same maturity in fatigue, processing, and regulatory approval, are almost entirely absent from this clinical distribution. The porous-architecture route came to dominate the clinic because it solves the modulus problem—by arranging a well-established material architecturally—without demanding a new alloy chemistry. None of this means β-Ti alloy research lacks value; it simply reflects that the two routes sit at different stages of clinical maturity (see Section 8).

## 8. Challenges, Future Directions, and a Proposed Standardization Checklist

Section 7 showed which failure modes the porous titanium architecture controls well in the clinic and where the laboratory–clinical gap persists. This section takes up the two structural problems that feed that gap—the regulatory framework and reporting inconsistency—and offers a concrete proposal to move the field forward.

### 8.1. Regulatory Framework

One of the biggest barriers to the clinical use of patient-specific 3D-printed implants is regulatory uncertainty. Under the European Union Medical Device Regulation (MDR 2017/745) [129], a load-bearing orthopedic implant falls into Class III, the highest risk class, which normally requires a notified body to review a comprehensive technical file together with clinical evidence. A key distinction, clarified in EU guidance (MDCG 2021-3) [130], is that additive manufacturing or adaptation to a patient’s anatomy does not by itself make a device “custom-made”: patient-matched devices produced within a validated design envelope follow the standard conformity-assessment route for their class, whereas custom-made devices—manufactured to a specific prescription for an individual patient—follow the procedure in MDR Annex XIII. Importantly, even custom-made Class III implantable devices are not exempt from notified-body involvement: in addition to the Annex XIII statement, their manufacturers must undergo a notified-body conformity assessment of the quality management system (MDR Annex IX Chapter I, or alternatively Annex XI Part A). Earlier accounts from the pre-MDR/transition period (e.g., Willemsen et al. [131]) described custom-device pathways in which a notified-body review was not required; under the MDR as now applied, this is no longer the case for Class III custom-made implants. The U.S. framework similarly separates “custom” devices (the FDA Custom Device Exemption) from “patient-matched” devices, which are regulated through the standard pathway for their device type; the FDA’s 2017 guidance “Technical Considerations for Additive Manufactured Medical Devices” stresses that AM devices face the same classification and submission expectations as their non-AM counterparts of the same type [57].

Within this framework, Willemsen et al. [131] successfully treated two emergency spine cases—kyphosis from neurofibromatosis type 1 and cervicothoracic dissociation from Gorham disease—with patient-specific, porous-tipped titanium implants. One important point is that, in the specific jurisdictional context Willemsen et al. describe, ethics committee review was not mandatory for custom-device use outside a clinical investigation; whether such review is required, however, varies substantially by country, institution, the device’s regulatory status, and whether data are collected for research, and it should not be treated as a general rule. In such cases the ethical responsibility rests with the physician, alongside the duty to prepare a comprehensive technical file and obtain informed consent. The workflow they describe—CT imaging → segmentation → design → finite-element analysis and compression testing → cadaver trial → direct metal printing → hot isostatic pressing → screw-hole finishing → polishing → sterilization—together with the importance of ISO 13485 certification, offers an institutionally reproducible model. Experience mattered: the process took about six months in the first case but only about six weeks in the second.

### 8.2. The Standardization Gap

The second structural problem behind the clinical–laboratory gap is inconsistent reporting and standardization. General additive-manufacturing standards exist (e.g., ISO/ASTM 52900 [10] for terminology and the wider ISO/ASTM 529xx series; the ISO 10993 series [14] for biological evaluation), and—contrary to the impression that biomedical AM is essentially unstandardized—material- and process-level standards specific to AM titanium and to additively manufactured implants have matured in recent years. ASTM F2924 [11] and ASTM F3001 [12] specify powder-bed-fusion Ti-6Al-4V and Ti-6Al-4V ELI, respectively (feedstock, chemical composition, microstructure, mechanical properties, and thermal treatment/HIP), and ISO 5092:2025 [13] sets out general principles for additively manufactured non-active implants, explicitly covering custom-made and patient-matched devices. What remains incomplete is, therefore, not process qualification or base-material specification but component-specific standardization: harmonized protocols for lattice fatigue, as-built architecture verification, physiologically representative multiaxial and corrosion-fatigue testing, and—above all—the linkage of manufacturing variables to clinical outcomes. Because differences in AM equipment, alloy composition, printing parameters, and post-processing can substantially change the performance of the final product, these component-level gaps still allow porous-implant studies to report porosity, pore size, fatigue performance, and clinical outcome metrics inconsistently—or not at all—undermining the comparability of studies and the assessment of what laboratory findings mean clinically. Ng et al. [132], addressing process, material, and regulatory considerations for 3D-printed medical devices together, likewise highlight the decisive importance of consistent powder-property and printing-parameter control and standardized characterization.

### 8.3. Proposed Minimum Reporting Checklist

As an original contribution toward closing this gap, we propose a minimum reporting checklist for porous titanium load-bearing implant studies (Table 5), aiming at consistent reporting at every link of the chain from material to clinical outcome so that the clinical correlate of laboratory claims can be tracked. Unlike the general additive-manufacturing, material, and biological-evaluation standards noted above (e.g., ISO/ASTM 52900 [10], ASTM F2924/F3001 [11,12], ISO 5092 [13], and ISO 10993 [14]), which specify process control, material specification, and biocompatibility testing in isolation, the checklist is organized around clinical failure modes: each reporting item is explicitly tied to the failure mode it informs (Section 7), so any laboratory parameter can be traced to its clinical consequence.

**Table 5:** Proposed (preliminary) minimum reporting framework for porous titanium load-bearing implant studies; each link of the manufacturing chain is connected to the related failure mode. This framework is put forward as a starting point for discussion and would require formal consensus development and validation before it could be adopted as a reporting standard. SLM: selective laser melting; EBM: electron beam melting; HIP: hot isostatic pressing; LoF: lack of fusion; PBS: phosphate-buffered saline; SBF: simulated body fluid; PROM: patient-reported outcome. A limitation of the framework should be stated alongside it. Several of the outcomes these items are intended to illuminate cannot be established by reporting alone: determining whether a given design will cause stress shielding requires measurement of the component’s structural stiffness, evaluation of its osseointegration performance, verification of the loading actually experienced in vivo, and the development and validation of predictive computational models. The items listed here are, therefore, the minimum documentation required to make such an evaluation possible and comparable across studies—a precondition for that work rather than a substitute for it. In the table, # denotes the item number and bold rows are reporting-category headings.

#	Item to Report	Related Failure Mode/Rationale
**A. Powder/material**		
1	Alloy grade and powder specification (composition, particle size distribution, sphericity, flowability)	Defect formation, biocompatibility (debris)
2	Powder reuse/batch number	AM reproducibility
3	Interstitial content: O, N, H	Mechanical properties and embrittlement
**B. AM process**		
4	AM method (e.g., SLM/EBM) and main parameters (power, scan speed, layer thickness, scan strategy/hatch spacing)	Microstructure, defects, surface quality
5	Build orientation	Anisotropy, fatigue
**C. Post-processing**		
6	Heat-treatment regime, HIP, and residual-stress assessment	Fatigue, distortion, debris
7	Surface post-processing (loose-powder removal, chemical/electrochemical/mechanical), cleaning validation, and sterilization method	Debris, surface chemistry, biological response
**D. Architecture/structural verification**		
8	Unit-cell type/topology; designed vs. measured pore size and strut diameter	Osseointegration, stress shielding
9	Porosity (%), strut thickness, interconnectivity by micro-CT; relative density; specific surface area	Real architecture verification, mechanical interpretation; infection-relevant surface exposure
10	Defect quantity: LoF, keyhole, gas pore	Fatigue, early fracture
11	Surface roughness: Ra/Sa/Sz	Osseointegration, debris, fatigue
**E. Mechanical/fatigue**		
12	Measured effective elastic modulus	Stress shielding/aseptic loosening
13	Component-specific fatigue testing: loading mode (compression/shear/bending/torsion/multiaxial), environment (air/PBS/SBF), R ratio, frequency, run-out cycles, normalized fatigue ratio	Lattice/strut fracture, corrosion-fatigue
**F. Surface/biological**		
14	Surface treatment and bioactive doping if any; coating method, thickness, and depth-uniformity of coverage across the porous region; evidence level (in vitro/in vivo/clinical)	Osseointegration, infection
**G. Clinical**		
15	Clinical data: study design, comparator, defect grade (e.g., Paprosky), follow-up, survivorship/endpoint definition, osseointegration criterion (e.g., Moore), PROM (e.g., HHS, pre-/postoperative)	Clinical validation of all modes
16	Retrieval (explant) analysis if available	Clinical debris/strut-fracture verification

Because ASTM F382 is specific to bone plates, an application- or component-specific equivalent fatigue protocol should be used for porous acetabular components, cages, cones, and augments, and the test conditions (item 13) should be reported explicitly.

To gauge how large the reporting gap actually is, we applied this 16-item checklist retrospectively to the clinical series summarized in Table 3, scoring each study for whether it reported each item (based on the data available in the source publications). Because the manufacturing- and architecture-side items (1–13) are structurally inapplicable to pooled analyses—a meta-analysis does not report powder reuse, interstitial content, or residual stress for its constituent studies—the two systematic reviews/meta-analyses (Almeida et al. [17] and Mancino et al. [18]) were excluded from item-level scoring, and the 12 primary clinical series were scored. A category-level summary of this scoring is provided in Supplementary File S1 (Table S1). The result illustrates the disconnect that motivates this review. The clinical outcome items (study design, follow-up, survivorship/endpoint definition, and PROMs; item 15) were reported almost universally, and the manufacturing method (item 4) was named in general terms where a specific process (SLM/EBM) was stated, whereas the full material/powder specification (item 1) and formal retrieval/explant analysis (item 16) were essentially never reported. By contrast, the powder-, post-processing-, and architecture-verification items were reported very rarely: interstitial content (item 3), powder reuse (item 2), residual-stress assessment (item 6), micro-CT porosity/interconnectivity and relative density (item 9), quantified LoF/keyhole/gas-pore defects (item 10), and surface roughness (item 11) were essentially absent across the clinical literature, and a measured effective elastic modulus (item 12) or a component-specific fatigue protocol (item 13) was almost never provided. In other words, the manufacturing- and architecture-side parameters that laboratory studies show to govern fatigue, osseointegration, and stress shielding are precisely the parameters that clinical series omit—so the laboratory–clinical link cannot be reconstructed retrospectively for most published cohorts. An important caveat qualifies this finding: for a clinical outcome paper, many manufacturing-side items (e.g., powder reuse, scan strategy, and residual-stress treatment) are not conventionally expected, because that information typically resides in the manufacturer’s technical file rather than in the clinical report. The low scores should, therefore, be read not as a criticism of individual clinical papers, which report what their scope requires, but as evidence that the manufacturing and clinical bodies of literature are documented separately and cannot currently be linked at the level of the individual implant. The checklist is, accordingly, intended not as a scorecard for past studies but as a prompt for future studies—and for manufacturer–clinician collaboration—to report the few manufacturing and architecture parameters that would make the laboratory–clinical link traceable. This scoring was carried out by the authors as an illustrative audit: each of the 12 series was scored against the 16 items from its source publication, with a study credited with an item only where that item was explicitly reported. Any items initially in doubt were resolved by re-checking the full text. This is a coarse, thesis-driven audit of the series already discussed rather than a validated instrument or a systematic screening exercise; even so, it converts the “reporting gap” from a qualitative impression into a concrete, itemized gap and defines exactly which items future studies would need to add.

### 8.4. Future Directions

The laboratory–clinical synthesis and the reporting framework developed above also point to the field’s next priorities. First and most urgent is standardizing fatigue testing: the field should move from today’s uniaxial-compression-dominated practice to component-specific protocols that mimic physiologically meaningful conditions—multiaxial, dynamic (compression–shear–bending–torsion) loading in a simulated-body-fluid (SBF/PBS) environment, with the R ratio, frequency, environment, and run-out cycle count reported explicitly and the normalized fatigue strength provided. Such conditions would also capture the corrosion-fatigue and fretting mechanisms that remain understudied in porous/additively manufactured titanium, closing the largest gap between laboratory data and in vivo reality. Second is the scalable production, in vivo validation, and integration into load-bearing implants of the bioactive surface strategies (Zn/Mg/Cu doping) that perform strongly in vitro—an avenue that could improve the infection and osseointegration modes at once. Third is making patient-specific design more accessible, both regulatorily and logistically. Materials science continues to offer promise too; machine learning approaches, for instance, are increasingly used to predict new titanium-alloy compositions with a low Young’s modulus and free of cytotoxic elements [133]. Overall, the route likeliest to grow clinical impact fastest in the short to mid term is maturing the fatigue, surface-functionalization, and standardized-reporting dimensions of the established porous Ti-6Al-4V architecture, while low-modulus β-Ti alloys remain a complementary long-term route as the barriers of manufacturability, fatigue, cost, and regulatory approval are overcome.

### 8.5. Converging Routes: Modern Alloys Within Porous Architectures

The two solution lines examined in this review are not mutually exclusive. The modern β-type and multi-principal-element (high-entropy) alloys still being developed in materials science could, in principle, be fabricated as porous, additively manufactured structures, stacking architectural and intrinsic alloy modulus reduction rather than treating them as alternatives—potentially reaching bone-matched stiffness at lower porosity, and, therefore, with less penalty to fatigue strength, than a porous conventional Ti-6Al-4V of the same effective modulus. Computational and data-driven alloy design is beginning to make such candidates tractable: machine learning models have been used to predict low-modulus, biocompatible titanium compositions [133], which could be screened for additive processability and then realized as porous scaffolds. Realizing these combined designs—supported by the failure-mode-linked standardized reporting proposed above, so that manufacturing and architectural variables can actually be compared across studies—is a natural direction in which modern alloy design and additive architecture may converge.

## 9. Limitations of This Review

The inferences of this study should be read in light of several limitations. First, it is a critical narrative review rather than a systematic one; the literature was searched in a targeted way, with no PRISMA-type quantitative flow. This provides flexibility for a thesis-driven synthesis but does not fully rule out selection bias. Second, the clinical evidence is heavily concentrated in hip-acetabular reconstruction; the evidence for spine, revision knee, and tumor/segmental reconstruction is more heterogeneous, involves smaller patient numbers, and is mostly evidence Level IV (case series) (Table 3). The conclusions drawn under the broad heading of “load-bearing orthopedic reconstruction”, therefore, cannot be generalized with equal confidence to every anatomical region.

Third, because most of the clinical series examined did not report manufacturing parameters (porosity, pore size, unit-cell type, and fatigue performance), the link between laboratory findings and clinical outcomes stays indirect in most places—precisely the gap the proposed reporting framework (Table 5) targets. The proposed checklist itself is a preliminary, non-validated instrument, and its retrospective application here was an illustrative audit performed by the authors—without formal inter-rater reliability testing or external validation—so the item-level pattern should be read as indicative rather than definitive. Fourth, central mechanical concepts such as stress shielding and fatigue were not measured directly in clinical series (e.g., serial DEXA/BMD or clinical fatigue monitoring), so the inferences about them rest largely on mechanistic, biomechanical, or preclinical evidence; likewise, much of the evidence on surface biofunctionalization and antibacterial strategies is at the in vitro or short-term animal level.

Fifth, the transparency of manufacturer-specific design and performance data for commercial implants is limited. Sixth, part of the materials-science layer—in particular several of the tabulated elastic-modulus values in Table 1 and a number of process–microstructure thresholds in Section 4 and Section 5—is drawn substantially from a single recent secondary review; where possible, we have anchored the key mechanistic claims to primary studies (e.g., the low-modulus mechanisms of β-Ti and Gum metal [28,29,30] and the SLM martensite and heat-treatment behavior [67,70]), but the individual tabulated values and some processing thresholds still rely predominantly on that secondary source and may deviate depending on primary measurement conditions. These should be read as representative literature values rather than independently pooled measurements. Seventh, several of the strongest acetabular series that carry the central thesis originate from overlapping author groups and institutions (e.g., References [2,20,21] share authors), which may limit the independence of the clinical evidence base and should be weighed when interpreting the pooled picture; how this overlap was identified and handled is set out in Section 2.2. Finally, since some of the 2025–2026 sources may still be in online-first/early-access status, final volume, page, and citation details should be updated at submission. None of these limitations invalidates the review’s central observation—that current clinical translation runs through the porous Ti-6Al-4V architecture—but they do underline that the evidence levels underpinning it must be carefully distinguished.

## 10. Conclusions

This review examined the role of porous titanium in load-bearing orthopedic implants through an often-overlooked translational gap. Over the past two decades, materials science has made notable progress in bringing the elastic modulus of β-type titanium alloys closer to that of bone. In clinical practice, however, the low effective modulus is achieved predominantly not through these alloys but through the porous architecture of well-established Ti-6Al-4V. The clinical evidence, synthesized along the axis of failure modes, shows that this architecture is associated with favorable short- to mid-term clinical performance, particularly low reported aseptic-loosening rates in acetabular cohorts—although all-cause revision-free survivorship remained more variable in complex revision cohorts, where infection, instability, and host-related factors dominate. It should be emphasized, however, that the reduction in stress shielding itself has not been directly measured in most clinical series; the link between porous architecture and reduced stress shielding rests largely on mechanical and finite-element evidence, whereas the low loosening rates are the clinically measured endpoint. In the dimensions of fatigue performance, antibacterial surface function, and reporting consistency, laboratory success has not yet found its clinical correlate.

The broader implication is this: Given the current evidence, the main priority for accelerating the clinical impact is not simply to develop new alloy chemistries but to make the established porous architecture fatigue-resistant, biologically functionalized, and consistently reported. This pattern reflects a difference in translational maturity rather than a judgment on the alloys themselves; the architectural route is the one currently in clinical use because it addresses the modulus problem with a material that already carries regulatory and clinical validation. It does not rule out a future role for low-modulus β-Ti alloys, which may enter clinical use more widely as they gain fatigue, manufacturability, and regulatory maturity. In this light, the proposed minimum reporting checklist (Table 5) is a concrete, immediately applicable step: by tying each design decision to its related clinical failure mode, it aims to improve the comparability of studies and the clinical traceability of laboratory claims and thereby help build the bridge among manufacturing, microstructure, surface function, fatigue strength, and clinical reporting on which the clinical promise of porous titanium ultimately depends.

## Data Availability

No new experimental or clinical data were generated in this review. The data produced are the authors’ own appraisals of the studies in Table 3—the illustrative reporting-checklist scores (summarized at the category level), the OCEBM 2011 level-of-evidence assignments, and the MINORS methodological appraisal—all of which are provided in machine-readable form in Supplementary File S1 (Tables S1, S3 and S4). No additional datasets were created or analyzed.

## Supplementary Materials

The following supporting information can be downloaded at: https://www.mdpi.com/article/10.3390/ma19183860/s1, Supplementary File S1 (an Excel workbook): A machine-readable version of the proposed minimum reporting checklist presented as Table 5 in the main text; the category-level retrospective application of the checklist to the 12 primary clinical series (Table S1); a key to those series (Table S2); the OCEBM 2011 level-of-evidence assignment for the clinical studies of Table 3 (Table S3); and the MINORS methodological appraisal of the 12 primary series (Table S4).
